# Deep Drug Discovery of Mac Domain of SARS-CoV-2 (WT) Spike Inhibitors: Using Experimental ACE2 Inhibition TR-FRET Assay, Screening, Molecular Dynamic Simulations and Free Energy Calculations

**DOI:** 10.3390/bioengineering10080961

**Published:** 2023-08-14

**Authors:** Saleem Iqbal, Sheng-Xiang Lin

**Affiliations:** Axe Molecular Endocrinology and Nephrology, CHU Research Center, Laval University, Quebec City, QC G1V 4G2, Canada

**Keywords:** SARS-CoV-2, pseudovirus ACE2 Inhibition, COVID-19, entry inhibitors, virtual screening, molecular dynamic simulations, MMGBSA calculations

## Abstract

SARS-CoV-2 exploits the homotrimer transmembrane Spike glycoproteins (S protein) during host cell invasion. The Omicron XBB subvariant, delta, and prototype SARS-CoV-2 receptor-binding domain show similar binding strength to hACE2 (human Angiotensin-Converting Enzyme 2). Here we utilized multiligand virtual screening to identify small molecule inhibitors for their efficacy against SARS-CoV-2 virus using QPLD, pseudovirus ACE2 Inhibition -Time Resolved Forster/Fluorescence energy transfer (TR-FRET) Assay Screening, and Molecular Dynamics simulations (MDS). Three hundred and fifty thousand compounds were screened against the macrodomain of the nonstructural protein 3 of SARS-CoV-2. Using TR-FRET Assay, we filtered out two of 10 compounds that had no reported activity in in vitro screen against Spike S1: ACE2 binding assay. The percentage inhibition at 30 µM was found to be 79% for “Compound F1877-0839” and 69% for “Compound F0470-0003”. This first of its kind study identified “FILLY” pocket in macrodomains. Our 200 ns MDS revealed stable binding poses of both leads. They can be used for further development of preclinical candidates.

## 1. Introduction

The COVID-19 pandemic is a tremendous threat globally with many variants arising, some of which are variants of concern (VOC), including omicron—B.1.1.529 [1]. Currently, there are several subvariants designated “Omicron,” including B.1.1.529, BA.1, BA.1.1, BA.2, BA.3, BA.4, BA.5, BF.7, BQ.1, XBB and more recently, Arcturus variant—XBB.1.16, a subvariant of the recombinant Omicron strain, that has been identified in 34 countries. As of July 2023, 216 countries have reported COVID-19 cases, with more than 768 million confirmed and approximately 6,952,522 deaths [2] (https://covid19.who.int/, accessed on 25 July 2023). More recently, Monkeypox virus and hemorrhagic fever virus (SHFV), which causes a lethal disease similar to Ebola virus disease, has also confronted global health emergencies. Although several therapeutic agents have been evaluated for treatment, no efficacious antiviral agents have yet been shown. The causative agent of COVID-19, SARS-CoV-2, causes a lower respiratory tract infection that can progress to severe acute respiratory syndrome and even multiple organ failure [3,4]. SARS-CoV-2 is an enveloped virus from the family Coronaviridae and genus beta-coronavirus, comprising a large positive-strand single-strand RNA (+ssRNA) genome (~30 kb), which encodes four structural proteins (spike, envelope, membrane, and nucleocapsid protein) that are components of the virus particle, 16 nonstructural proteins (Nsp), mostly with enzymatic activities, and 6 accessory proteins [5,6,7]. Receptor binding is a key step of virus invasion [8]. Similar to severe acute respiratory syndrome coronavirus (SARS-CoV), SARS-CoV-2 uses its spike (S) protein to recognize the host receptor ACE2 [9]. The SARS-CoV-2 utilizes ACE2 as the receptor for entry into target cells [10]. Therefore, the S protein determines the infectivity of the virus and its transmissibility in the host [11]. Very recently, Carabelli et al. (2023) [12] have presented a comprehensive analysis of the immune escape, transmission, and fitness characteristics of SARS-CoV-2 variants.

Differential Sensitivity of the Natural Variants and Experimental Mutants to a Panel of Convalescent Serum Samples is shown in Figure S1A. Moreover, the latest COVID-19 research findings reveal that XBB.1 exhibited 30-fold resistance against breakthrough BA.2 infection sera and significant resistance against BA.2 infection sera [13].

SARS-CoV-2 keeps evolving into new variants due to sustained global transmission [14]. Furthermore, none of the variants and mutants demonstrated significantly altered sensitivity to all 10 convalescent sera, i.e., the EC50 values were not altered by more than fourfold, irrespective of an increase or decrease, when compared with the reference strain (Figure S1B). The point mutation-induced structural flexibility in S-protein, D614G, shifts the conformation of the S protein towards ACE2- binding fusion competent state and, hence, enhances SARS-CoV-2 infectivity in human lung cells [15]. SARS-CoV-2 macrodomain within the nonstructural protein 3 counteracts host-mediated antiviral adenosine diphosphate–ribosylation signaling. The catalytic mutations render viruses nonpathogenic, thus, making this enzyme an antiviral target. Taking such high data into consideration, with less impact of the mutation on RBD, we proceeded with nsp3 studies. COVID-19 can be controlled by designing small molecule drugs or monoclonal antibodies based on the process of viral binding to cell receptors. Due to the continuous emergence of new virus mutants, more drugs need to be screened.

**Figure 1:**
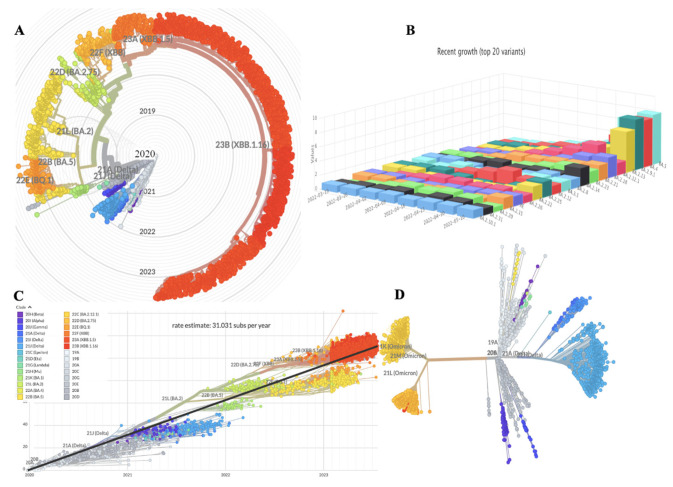
Illustration of genomic epidemiology of 2844 SARS-CoV-2 genome: (**A**) Phylogenetic tree with Radial Clade depicting the SARS-CoV-2 Variants of Concern (VOC). (**B**) 3D representation depicting time changes in the no of observations of SARS-CoV-2 throughout the world. (**C**) Phylogenetic tree depicting the Genomic epidemiology of SARS-CoV-2 represented by clock clade. (**D**) Rooted phylogenetic tree depicting the Genomic epidemiology of SARS-CoV-2.

The radial, rooted Phylogenetic Tree (PT) depicting the genomic epidemiology of 2844 SARS-CoV-2 genomes sampled between Dec 2019 and July 2023 is shown in Figure 1A, and Figure 1B represents the time changes in the number of observations of SARS-CoV-2 throughout the world. Phylodynamics analysis of the viral genome as interpreted in Nextstrain presents a real-time view into the evolution and spread of a range of viral pathogens of high public health importance [16]. PT depicting the Genomic epidemiology of SARS-CoV-2, represented by clock clade and rooted one, are displayed in Figure 1C,D respectively.

**Figure 2:**
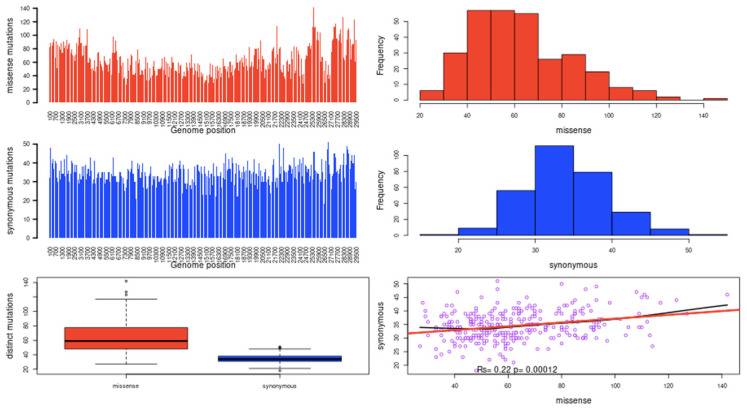
Variation Distribution plot of nsp3 depicting the Genomic distribution of Missense and synonymous mutations.

Nsp3 is the largest multidomain protein (~200 kDa) in coronaviruses and is notable because of the presence of a key enzyme, papain-like cysteine protease (PLpro), which is essential for viral replication and a target protein for drug discovery [17,18,19]). The Nsp3 is found to be significantly different in two SARS-CoVs in comparison with other Nsps [20,21]. The SARS-CoV-2 nsp3 includes three tandem macrodomains (Mac1, Mac2, and Mac3) [22]. The Genomic distribution of Missense and synonymous mutations of nsp3 is shown in the variation distribution plot in Figure 2.

Mac1 is present in all CoVs, unlike Mac2 and Mac3, and early structural and biochemical data demonstrated that it contains a conserved three-layered α/β/α fold and binds to mono-ADP-ribose (MAR) and other related molecules [23,24,25,26,27,28]. Macrodomains specifically recognize these modifications and serve therefore as “reader domains” of this posttranslational modification [29]. Computational studies have shown that coronaviral Nsp3 may comprise 10–16 domains [30,31]. SARS-CoV-2 Nsp3 also includes multiple domains, an N-terminal ubiquitin-like (Ubl) domain followed by a highly variable and conserved macrodomain that binds to ADP-ribose (ADP) [32]. It has recently been proposed that de-mono-ADP-ribosylation of STAT1 by ADRP may be linked to the Cytokine Storm Syndrome that is commonly observed in severe cases of COVID-19 [33]. Computational studies have been significant in identifying potential inhibitors of SARS-CoV-2 RdRP [34].

SARS-CoV-2 encodes the macrodomain (Mac1) domain within the large nonstructural protein 3 (Nsp3), which has an ADP-ribosylhydrolase activity conserved in other coronaviruses. In the context of reciprocal investigations, the binding affinities of CoV Mac1 proteins with ADP-ribose were observed within the range of 7 to 16 mM. Conversely, human Mdo2 exhibited a markedly enhanced binding affinity, surpassing the viral macrodomains by a minimum of 30-fold, with an affinity of 220 nM (Figure S2A,B) of the referenced publication [23]. Utilizing a thermal shift assay as an alternative approach to validate ADP-ribose binding, all four macrodomains subjected to assessment displayed elevated denaturation temperatures upon the introduction of ADP-ribose, as shown in Figure S2C.

Therefore, currently SARS-CoV-2 Mac1 enzyme is considered an ideal drug target and inhibitors developed against them can possess a broad antiviral activity against CoV. Considering this, the ADP-Ribose-1′’-phosphate-bound closed form of Mac1 domain is considered for screening with large commercial databases like Spec, Asinex, Life chemicals, ZINC, etc., which have been widely used for structure-based virtual screening [19,35]. We applied Extra Precision (XP) docking and Quantum polarized Ligand Docking (QPLD) providing strong potential lead compounds that perfectly fit inside the binding pocket. QPLD algorithm begins with a Glide docking job that generates several geometrically unique protein–ligand complexes. Glide poses are subjected to charge refinement using the QM/MM method (Schrodinger release 2021-4). A convolutional deep neural network-based approach called ‘Docking decoy selection with Voxel-based deep neural nEtwork’—(DOVE) [36] was used for selecting the protein model for docking (See Supplementary Information). Trained on two million structures, DOVE considers atomic interaction types and their energetic contributions as input features applied to the neural network. Using DOVE, we applied this convolutional neural network model to Crystal structure of SARS-CoV-2 spike receptor-binding domain with ACE2 (PDB entry 6M0J), SARS-CoV-2 macrodomain in complex with ADP ribose (PDB entry 6WOJ), (Table S1) and considering correct and acceptable according to CAPRI, a decoy of high probability (>0.5) and very small probability (<0.01). The term “Deep” in the manuscript title represents the DOVE method applied in our modelling studies.

Moreover, Laurini et al., performed computational-based simulations of the interaction between S-Protein region—receptor-binding domain (S-RBD) of SARS-CoV-2 and Angiotensin-Converting enzyme 2 (ACE2), highlighting the residues that play an important role across human receptor/viral protein binding interface [37]. During the course of our research investigations, a concurrent scientific inquiry conducted by Han et al. (2022) confirmed that Spike proteins from Omicron, Delta, and the prototype SARS-CoV-2 exhibit comparable affinity for binding to human angiotensin-converting enzyme 2 (hACE2) [38]. In a recent study, it was elucidated that monoclonal antibodies targeting the receptor binding motif were ineffective in neutralizing the Omicron variant [39]. Building upon this noteworthy finding, we proceeded to evaluate the efficacy of various compounds in inhibiting the interaction between the wild-type Spike protein and ACE2 receptors. Hence, this manuscript aims to undertake a parallel investigation into potential inhibitors of the SARS-CoV-2 Spike protein, utilizing an experimental Pseudovirus ACE2 Inhibition TR-FRET Assay.

## 2. Materials and Methods

All calculations reported in this work involved various methods. All methods were carried out in accordance with relevant guidelines and regulations. For evaluating protein docking for SARS-CoV-2 models, we used a convolutional deep neural network-based approach (Refer Supplementary Data methods). For in vitro screening against Spike S1: ACE2 binding assay, we used ACE2 Inhibition TR-FRET (Time Resolved Forster/Fluorescence energy transfer) Assay. For the solvation free energy calculations between different ligand bound complexes, we used MMGBSA calculation and its implied mathematical equations. For extended assay conditions, the list of compounds and their test range used in this study for WT Spike: ACE2 Binding, data analysis, molecular descriptors and all other details relevant to our SARS-CoV-2 study have been reported in the Supplementary Section. All data generated or analysed during this study are included in this published article (and its Supplementary Information Files).

### 2.1. Mutational Landscape

Mutational studies were carried out in an elucidated way by (Li Q et al., 2020) [40], who analysed 80 natural variants and 26 glycosylation spike mutants using a pseudovirus assay. The group concluded glycosylation deletions were less infectious.

### 2.2. Mac 1 Hydrolytic Activity and Cell Culture Experiments

Cell culture experiments have revealed that SARS Mac1 is dispensable for viral replication in some cell lines [41,42,43,44] (Fehr et al., 2016, Erikson et al., 2008, Putics A et al., 2005, Fehr et al. 2014). In addition to the animal studies reciprocating that Mac1 hydrolytic activity promotes immune evasion and that it is essential for viral replication and pathogenicity in the host [45], targeting Mac1 may also have the benefit of enhancing the innate immune response, as Mac1 is required for some corona viruses to block interferon (IFN) production.

### 2.3. TR-FRET (Time Resolved-Forster/Fluorescence Energy Transfer) Assay

FRET assays utilize donor fluorophores with longer emission times (1–2 ms), such as lanthanide ion complexes, to reduce potential interferences caused by the excitation energy. The optimal distances between donor and acceptor fluorophores are similar to FRET pairs. As TR-FRET assays utilize a time delay between fluorophore excitation and signal acquisition (approximately 50–150 μs in duration) and this acquisition delay is sufficient for avoiding interference by the usual short-lived fluorescence from test compounds.

### 2.4. Experimental Conditions

#### 2.4.1. ACE2:SARS-CoV-2 Spike Inhibitor Screening

The ACE2:SARS-CoV-2 Spike Inhibitor Screening Assay Kit is designed for screening and profiling inhibitors of this interaction namely, ACE2, His-Tag, Eu-labeled (BPS Bioscience, #100705, San Diego, CA, USA), SARS-CoV-2 Spike S1 Protein, biotin-labeled (BPS Bioscience, #100720), 3X ACE2: Spike TR-FRET Buffer (BPS Bioscience, #79963) and Dye-labeled acceptor (BPS Bioscience). The enzyme and substrate concentration used are shown in Supplementary Section—Table S2.

#### 2.4.2. Assay Conditions

Five µL of ACE2-Eu were incubated with 5 µL of dye-labeled acceptor and 5 µL of inhibitor (see Table S3). Binding reaction was started by the addition of 5 µL of Spike S1-Biotin, as described in the protocol kit #78281 (Spike S1 [Wild-Type] [SARS-CoV-2]: ACE2 TR-FRET Assay Kit). The reactions were incubated for 1 h at room temperature, and the TR-FRET signal was finally measured in an Infinite M1000 microplate reader (Tecan, PerkinElmer, Waltham, MA, USA) at excitation of 340 nm and emissions at 620 nm and 665 nm.

### 2.5. Data Analysis

Binding assays were performed in duplicate at each concentration. The TR-FRET data were analyzed using Prism (GraphPad, San Diego, CA, USA). Percent inhibition was determined by normalizing the data to signal from negative control wells (Blank, wells without biotinylated ligand, set as 100% inhibition) and positive control wells (No compound, wells in the absence of any inhibitor but with respective buffer, set as 0% inhibition). Data for a reference inhibitor, Anti-Spike, were included as a control for inhibition. The values of percentage activity were plotted on a bar graph.

### 2.6. Molecular Dynamics Simulation (MDS)

To understand the dynamic behavior of the docked complex of the macrodomain and the top leads, molecular dynamics (MD) simulations of 200 ns (nanoseconds) run for each complex were carried out using the Desmond version 4.4 (Schrodinger suite) after carrying out the molecular modelling of different complexes with active site-bearing pocket residues, viz., Ile23, Val49, Gly51, Ala52, Pro125, Leu127, Ser128, Ala129, le131, Phe132, Val155 and Phe156. Protein was set in water for solvation using the TIP3P box. Following solvation, charge neutralization was carried out by the addition of Na+ and Cl^-^ ions [46]. Neutralization was carried out by adding 3 Na+ ions. Salt concentration was set to 0.15 M sodium and chloride ions to approximate physiological condition. Force field OPLS5 was set, and the grid dimensions were parameterized with an internal size of 10 Å 10 Å 10 Å (x Å y Å z Å). Minimization was carried out for the total simulation box in volume (Å^3^) 253,786, which contained protein, water, and ions for 2000 steps using the steepest descent algorithm and then for 3000 steps using the conjugate gradient [47]. The structure was analyzed for its stability and potential energy after the completion of the steps. The total system was then equilibrated. The simulation was performed under the conditions of constant temperature and pressure as the protocol for simulations as defined by [48] was followed. Briefly, the Martyna–Tobias–Klein scheme was used for pressure coupling. PME algorithm [49,50] was used for calculating the electrostatic forces. The particle mesh Ewald was utilized to manage the long-range electrostatics, with a relative tolerance between long and short-range energies of 1 × 10^−9^. A real-space cut-off of 9 was used to analyze short-range interactions; all runs were performed at 300K at constant volume and temperature (NPT ensemble) under certain periodic boundary conditions in order to achieve a completely converged system for 200 production runs. MD simulations were carried out by using the workstation-HP Z4 Generation 4 Z-Platform W2235, with ‘GeForce RTXTM 3070 Ti and RTX 3070 graphics card’, available at Dr. Lin’s Computational Biology Lab, Chu de Quebec Hospital, Quebec City, QC, Canada.

### 2.7. Binding Affinity Analysis

The energy of the interaction was calculated from the snapshots taken from the simulation run. Solvation free energy and ∆G_vacuum_ were also calculated and compared between the different ligand-bound complexes. Higher binding affinity in complexes might be attributed as Omicron Spike Protein has a Positive Electrostatic Surface that Promotes ACE2 Recognition. In contrast to the alpha, beta, and gamma variants, the omicron variant’s RNA Binding Domain (RBD) exhibits a comparable affinity to the prototype RBD when binding to hACE2. This similarity in binding affinity might be attributed to the presence of multiple mutations that potentially compensate for both immune evasion and enhanced transmissibility.

Interaction distances were used to gauge the binding enthalpy of the s-protein to ACE2 system. These distances were defined as the distance between the centers of the intermolecular bond donor and acceptor heavy atoms. The binding entropies were not directly calculated due to the inherent difficulties achieving “convergence” of these calculations on such large systems. It should be noted that, for an accurate calculation of the s-protein binding entropy, the full s-protein trimeric construct would need to be simulated (not just the RBD) and the complete receptor bound complex would also include membrane models to anchor the host/viral proteins.

### 2.8. MMGBSA Calculations

MMGBSA method [51] was employed to calculate the free energy and energy decomposition per residue, and the ΔG_bind,solv_ was calculated. Such computational calculations have been used recently in identifying the PLpro-SARS-CoV-2 inhibitors [52]. For calculating the ligand binding energies and ligand strain energies for docked complexes of leads, we used the Prime module of Schrodinger, which was set to (i) Variable-dielectric generalized Born Model (VGSB) and which uses water as continuum solvation model for refinement, (ii) OPLS3—force field, and (iii) minimization sampling method which minimizes all-atom in each residue. Binding free energies and prediction of reliable ligand binding poses consist of performing multiple short MD simulation replicas, wherein the last 25 ns (175–200 ns) and 100 frames over this ensemble were taken into consideration using embedded python script in Desmond, viz., thermal_mmgbsa.py. The energy of the interaction was calculated from the snapshots taken from the simulation run. Solvation free energy and ΔG_vacuum_ were also calculated and compared between the different ligand-bound complexes. For the free energy calculation, the following equations were used:

ΔG_binding_ = G_complex_ − G_receptor_ − G_ligand_

ΔG_Tot_ = ΔG_gas_ + ΔG_solv_

ΔG_gas_ = ΔE_ele_ + ΔE_vdw_

ΔG_solv_ = ΔE_polar-solvation_ + ΔE_non-polar_

Implying

ΔG_bind_ = ΔE_MM_ + ΔG_SOL_ + ΔG_SA_

where ΔE_MM_ is the difference in the minimized energies between the mac domain-inhibitor complex and the sum of the energies of the unbound mac domain and inhibitor. ΔG_SOL_ is the difference in the GBSA solvation energy of the protein–inhibitor complex and the sum of the solvation energies for the unbound mac and inhibitor. ΔG_SA_ is the difference in surface area energies for the complex and the sum of the surface area energies for the unbound mac domain and inhibitor.

## 3. Results

### 3.1. Binding of RBD of the SARS-CoV-2 Spike Protein to ACE2 Monitored by TR-FRET Assay

Time-resolved FRET (TR-FRET) assays are increasingly used to monitor molecular interactions at nanometer scale with a high signal-to-noise ratio due to temporal separation between sample excitation and energy transfer measurements. TR-FRET combines proximity features of FRET assays with time-resolved fluorometry (Refer TR-FRET assay Supplementary Section). Pseudovirus ACE2 Inhibition TR-FRET Assay was carried out to check inhibitory effects of compounds against Spike: ACE2 binding (Refer to Supplementary Information for experimental conditions (Table S2) and materials and methods). For a list of ten TR-FRET assayed compounds and their test range used in this study, see WT Spike:ACE2 Binding assay (Table S3). Reactions were incubated for 1 h at room temperature, and TR-FRET signal was finally measured in an Infinite M1000 microplate reader (Tecan) at excitation of 340 nm and emissions at 620 nm and 665 nm (Supplementary protocol).

### 3.2. Inhibition of SARS-CoV-2 Spike RBD Binding to ACE2

**Figure 3:**
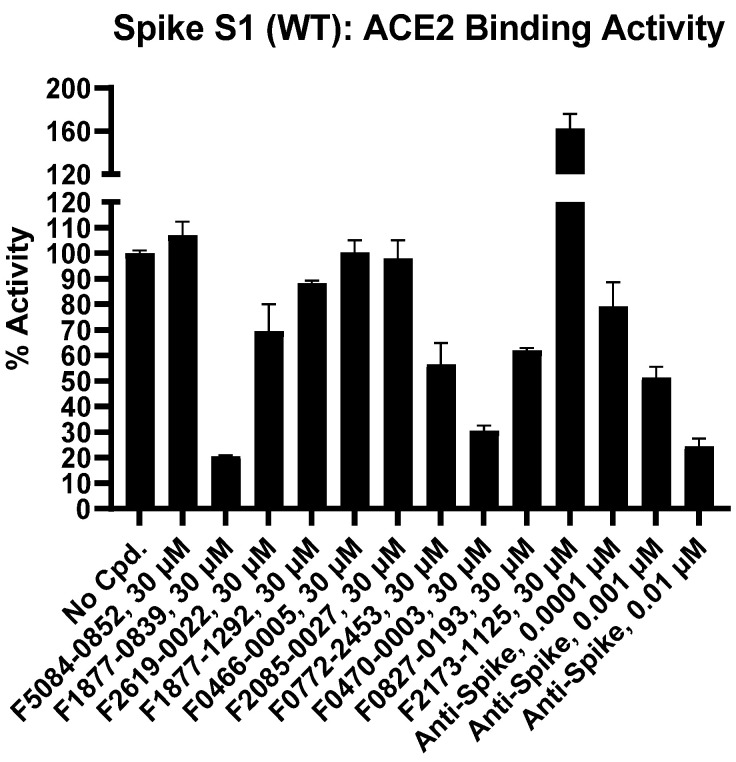
Percentage activity of compounds against Spike S1 (WT): ACE2 Binding.

Percentage inhibition is summarized in Table 1 and Table 2. Moreover, data for a reference inhibitor, Anti-Spike (0.0001 µM, 0.001 µM, 0.01 µM concentrations), were included as a control for inhibition. The values of percentage activity of compounds against Spike S1 (WT): ACE2 Binding were plotted on a bar graph (Figure 3). “Compound F2173-1125” was brightly coloured in the assay experiment and led to interference with TR-FRET signalling. At 30 µM concentration, percentage inhibition for all compounds revealed “F1877-0839” as the most potent inhibitor and showed 79% inhibition, which could be ascribed due to its possession of ribose scaffold moiety.

## 4. Result and Discussion

Motivated by the fact that macrodomain can be inhibited by multiple drug-like ligands, and the drugs acting at protein–protein interfaces on spike protein [53]) and less-studied protein/domain, viz., Mac1 domain of Nsp3 reducing viral entry to a mammalian host cell. We speculated that a range of drug molecules may efficaciously interact with ADP binding pocket. The discovery and characterization of macrodomain inhibitors have been described here. To design ligands with high specificity and affinity, it is crucial to understand structural determinants for protein–ligand complexes at an atomic level. We carried out all-atom MDS run for 200 ns and free energy calculations for the top two hits that are widely used in studies of biomolecules, guided with fast and efficient deep learning (for selecting the target) and QPLD, which provides change in angle conformations that bound more accurately inside the binding pocket. In addition to identification of several high-potency drugs and/or molecules, we unveiled consensus-binding mechanism that the oxazole ring of both the drugs prefers to bind to core residue anchoring Phe 156, a residue novel to the SARS-CoV-2 virus family. Ligands anchoring at this site might facilitate future design and optimization of an inhibitor for the SARS-CoV-2.

In our modeling studies, to acquire comprehensive insight into a putative inhibitor, a three-step docking procedure using molecular docking filters such as (HTVS, XP docking) was used. Figure S3 represents the two-dimensional chemical structure representation of identified compounds used in study. After an in-depth analysis of binding patterns, the third step in terms of induced fit docking (flexible) was employed. The glide docking of Schrödinger LLC Maestro package (New York, NY, USA) was initially carried out using Standard Precision (SP docking) followed by the scoring function of “extra precision” (XP) Glide. XP takes care of (i) large desolvation penalties to both ligand and protein polar and charged groups in appropriate cases and (ii) identification of specific structural motifs that provide exceptionally large contributions to enhanced binding affinity. Results were ranked by docking scores and were found to be −12.87, −9.61 and −11.19 kcal/mol for (F1877-0839), (F0470-0003) and cocrystal-docked complexes, respectively (Table S4). Inhibitory activity profound in compounds “F1877-0839” and “F0470-0003” may be attributed due to the presence of a thiazole ring in both compounds. Visualization of best post-docking poses of the drug-target receptor complexes in terms of hydrogen bonding, hydrophobic and π–π interactions were analyzed using Chimera visualization tool. Three-dimensional images were drawn using Pymol version 1.3 The PyMOL Molecular Graphics System.

**Figure 4:**
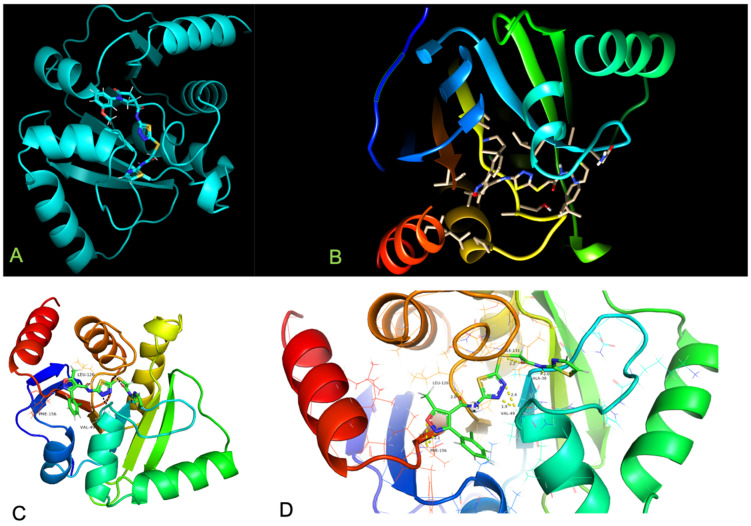

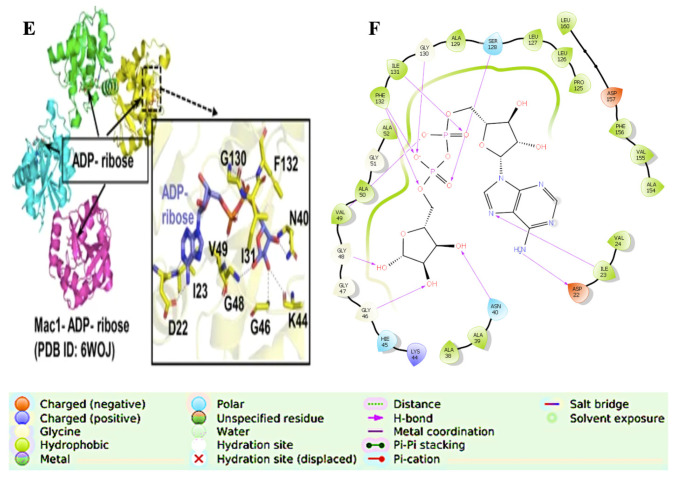
Docked complexes of “F1877-0839” and “F0470-0003” and their binding modes disclosed in the ribbon model. (**A**) Cyan representation of docked complex of “F1877-0839” (**B**) Docked complex of “F0470-0003”. (**C**) Hydrogen bonding interactions of compound “F1877-0839” at binding pocket of the macrodomain-ACE2 interface. (**D**) Zoomed in catalytic site displaying key hydrogen bonding residues especially Phe156 and molecular interaction with subsites in the protein–ligand docked complex. (**E**) ADP-ribose bound at catalytic site, while (**F**) represents the 2D interaction showing various molecular interactions of the cocrystal.

The docked-ligand molecule interaction within subsites is shown in Figure 4A,B). Adenine moiety is sandwiched between α2 and β7 in a mostly hydrophobic environment created by Ile23, Val49, Pro125, Val155, and Phe156. Polar contacts are facilitated by Asp22, which forms a hydrogen bond to N6 atom via its carboxylate group, and by the main-chain amide of Ile23, which binds to N1 atom. A striking insight in our study is that residue Phe156 is replaced by Asn in closest homologues from SARS-CoV and MERS-CoV. Phe156 is one of important residues in SARS-CoV-2, and in our simulation studies, lead inhibitors show consistent hydrogen bonding with such residue, Phe156, revealing the potency of the ligand-bound protein complexes of (F1877-0839) and (F0470-0003) (Figure 4C,D). As depicted in Figure 4E,F, the diphosphate moiety forms hydrogen bonds with key interacting residues Ser128 and Ile131. Phe132, Leu127, Ala129, Ala52, and Gly51 pave the way for hydrophobic interactions. Scaffold development of above-mentioned two inhibitors bound very well at the Mac1 active site providing critical structure–activity data for inhibitor optimization and our analysis reveal a path for accelerating the development of such inhibitors as potential candidates for antiviral therapeutics.

As MDS complements experimental research and provides structural information at the atomic level with dynamics without facing the same experimental limitations, MDS is an excellent tool for carrying out in silico investigations in drug discovery and other scientific research.

We first equilibrated crystal structure of SARS-CoV-2-ACE2 [Protein Data Bank (PDB) entry 6WOJ] in physiologically relevant environment. MDS was performed as previously described with slight modifications. MDS for 200 ns was run for the final equilibrated structure (Supplementary Simulation methods). Furthermore, to understand stability of the ligand in the binding pocket of the macrodomain, RMSD plots for the backbone atoms for both protein and ligand-bound protein complexes of (F1877-0839) and (F0470-0003). All runs for MD simulations of nsp3 macrodomain–ligand-bound complexes have been performed at the normal temperature. Structural deviations of each protein–ligand complex in terms of RMSD, which characterizes conformational stability in protein Cα backbone, RMSF for checking residual fluctuation cum flexibility, and rGYR for compactness of each complex system, were investigated during MDS run of 200 ns. The stability of protein–ligand complexes was gauged during 200 ns. Ligand properties in terms of PSA, SASA, and rGYR illustrating the stability of the macrodomain best inhibitors (F1877-0839) and (F0470-0003), respectively, into the protein-binding pocket are displayed in Figure S4. For simulated docked complex containing compound (F0470-0003), average RMSD of protein Ca-backbone, which was of magnitude of (0.6 Å), attained a steady state after 100 ns simulation run, while compound (F1877-0839) remained steady for the initial 50 ns with deviation of 1 Å, and for the remainder 150 ns, showed an average RMSD of 1.2 Å (Figure S4). The average values for RMSF as well as rGYR are reported in (Figure S4), and our overall simulation study reveal the stability of both the simulated complexes, signifying the binding mode at the catalytic site of the macrodomain.

**Figure 5:**
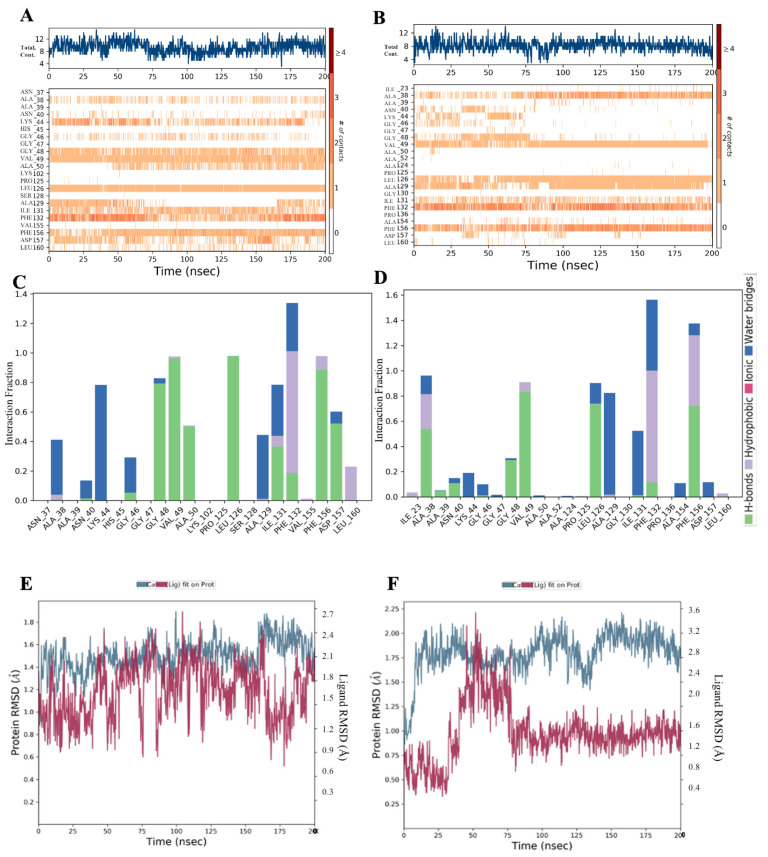
(**A**,**B**) Molecular interaction diagrams showing the consistency of hydrogen bonds of the key interacting residues. (**C**,**D**): Macrodomain (Mac) Catalytic site residues and active site hydrogen bond networks fractions of (F1877-0839) and (F0470-0003). (**E**,**F**): Corresponding RMSD’s of the protein Ca-backbone along with respective ligands F1877-0839 and F0470-0003.

Molecular interaction diagrams can be evidenced in terms of histograms and plots shown in Figure 5, where residues Ala38, Gly48, Val49, Leu126, Ala129, Ile131, Phe132, and Phe156 contributed to hydrogen bonding interactions throughout the 200 ns simulation run. However, residues Val49, Phe132 and Phe156 interacting were more energetically favourable, consistent in terms of maintaining the hydrogen bond contact, and played an important role in the regulation of binding the drugs at the catalytic site, paving the way for such class of compounds as potent inhibitors. These results reveal the potency of identified inhibitors for designing of potential therapies targeting this unique highly conserved protein domain.

Furthermore, to exemplify the conformational changes of every rotatable bond of both drug leads, the torsional profile was calculated during a simulation run of 0.00 to 200.00 ns. Figure S5A,B represent probability density of torsion of (F1877-0839) and (F0470-0003) illustrated in terms of dial plots; the data so obtained have been plotted on bar plots (histograms) and provide insights into conformational strain ligand that undergoes to maintain a protein-bound conformation. Additionally, the protein RMSF is useful for characterizing local changes along the protein chain has been shown (Figure S5C,D). The docked complex of Compound ID: F1877-0839 and Compound ID: F0470-0003 WT Spike: ACE2 Binding (A) labelled with interacting residues (B) without interacting residues are illustrated in Figures S6 and Figure S7, respectively.

To retain best compounds initially, filters like Rule of Five, Drug Likeness, and ligand-based Absorption, Distribution, Metabolism, and Excretion/Toxicity prediction (ADMET) profiling aids in decreasing potential risks during clinical development (Table S5), and other molecular descriptors were taken into consideration. Interestingly, RMSD of the protein’s backbone calculated against the crystal (initial) one (PDB entry 6WOJ) saturated at 1.7 Å after ∼10 ns, revealing the stability of the complex. One caveat to our study is that we used monomeric forms of RBD and ACE2 for MDS, which revealed that the Spike protein was highly unstable. The native Spike protein is a trimer and has several other domains, including the nearby N-terminal domain, and the native ACE2 protein can exist as a dimer.

Conformational dynamics analysis of the structural stability of complexes throughout 200 ns molecular dynamic simulations were estimated by calculating the protein–ligand stability in terms of RMSD. RMSD of the protein provides us insights into its structural conformation throughout the simulation. RMSD analysis discloses if the simulation has equilibrated. Both the simulated complexes were subjected to stability analysis. As shown in Figure 5E,F, displays corresponding to RMSD’s of the protein Ca-backbone along with respective ligands F1877-0839 and F0470-0003 (Supplementary Section for conformational Dynamics). This molecular dynamic study further confirmed our TR-FRET assay where inhibition of the drug (F0470-0003) was less than the (F1877-0839), due to much conformational change in the aforementioned drug, thus imparting its low stability than the potent drug-(F1877-0839) attaining high stability.

### Spike Protein Has a Positive Electrostatic Surface That Promotes ACE2 Recognition

**Figure 6:**
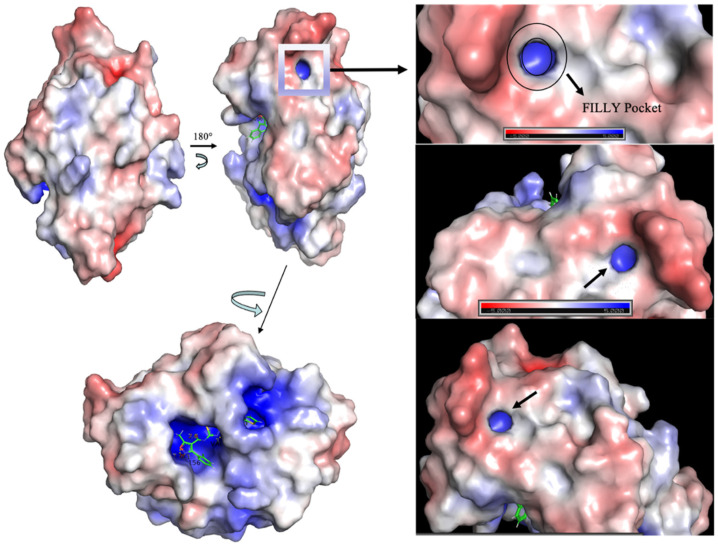
Electrostatic surface representation of the macrodomain-bound compounds at its catalytic site. Small single-direction arrows disclose the novel pocket; FILLY pocket.

The use of electrostatic potential surfaces is a common approach for mapping complementary interaction interfaces in biomolecular complexes (Weiner et al., 1982; McCoy et al., 1997) [54,55]. We used structural modeling to demonstrate that the macro domain is attracted to ACE2 receptors by long-range electrostatic forces leading to efficient recognition of the receptor (Figure 6). Our results agree with recent studies by [56] for the gradual increase of +ve charge for emerging variants, including higher lineages of omicron. The catalytic site of the macrodomain as per electrostatic calculations is concerned (Figure 6) when the ligand binds were rotated to 180°, the surface is red showing its high +ve charge. To our knowledge, our mapping identified a novel circular pocket, highlighted with the dark circle (Figure 6) formed by residues Ser 5, Phe(F)6, Tyr(Y)8, Leu(L)11, Ile(I)17, Val29, leu136, Leu138, and Tyr 150, when rotated translationally. We coined the pocket with a single-letter amino acid name and designated it as the “FILLY” pocket. Residues surrounding the pocket are Arg 139 and Val 149. A short black arrow pointing toward the enclosed site in Figure 6 discloses the identified pocket. Do such residues in the “FILLY” pocket represent any allosteric site or aid in glycosylation or represent a channel? This needs to be scientifically investigated to understand its biological significance.

Interestingly, as shown in the inset of Figure 5A–D, we found that during the equilibration, significant role of branched-chain amino acids such as Valine (Val), Leucine (Leu), Isoleucine (Ile), and aromatic amino acids, viz., phenylalanine (Phe), whose levels are high in COVID-19 hospitalized patients, such residues became equilibrated during molecular dynamics simulations, aiding us in reciprocating our identified leads as potent inhibitors.

## 5. MMGBSA Calculations

**Figure 7:**
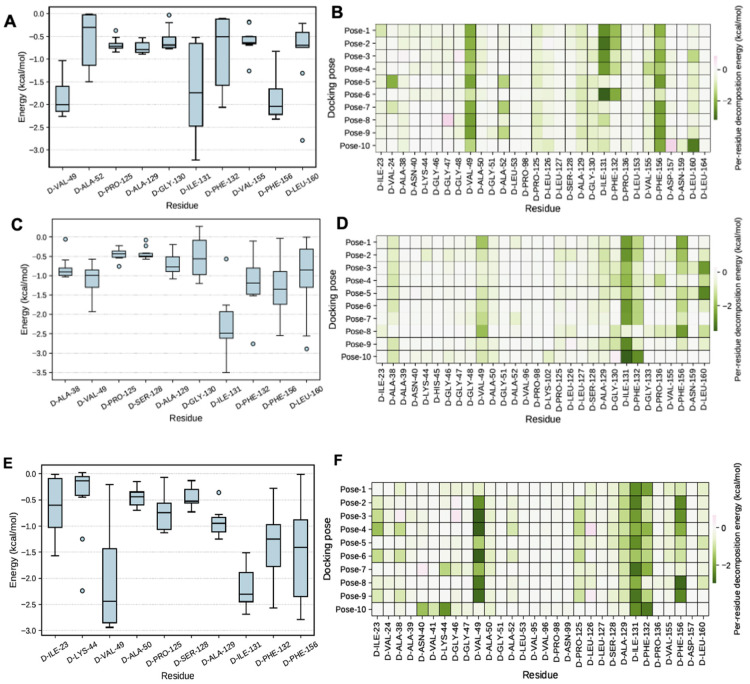
(**A**,**B**) per residue energy decomposition of (F1877-0839)-mac1 complex and MM/PB(GB)SA computation of the key interacting residues. (**C**,**D**): per residue energy decomposition of (F0470-0003)-mac1 complex and MM/PB(GB)SA computation of the key interacting residues. (**E**,**F**): per residue energy decomposition of (cocrystal)-mac1 complex and MM/PB(GB)SA computation of the key interacting residues.

It is of great interest in modern drug design to accurately calculate the free energies of protein–ligand or nucleic acid–ligand binding. MM-PBSA (mol. mechanics Poisson-Boltzmann surface area) and MM-GBSA (mol. mechanics generalized Born surface area) have gained popularity in this field. Structure-truncated MM/PB(GB)SA free energy calculation, and per-residue energy decomposition based on multiple poses have been accessed using the fastDRH tool (Wang Zhe et al., 2022)., which predicts the ligand binding mode and the hotspot residue of protein for ligand binding. The protein–ligand complex interaction is represented in Figure 7A–F.

To calculate free energy and energy decomposition per residue and the ∆ G_bind,solve_ MMGBSA calculations (Supplementary Section for the methodology and the binding affinity). In Table 3, compound F1877-0839 showed higher binding free energy (∆G _Total_ = −106.38 kcal/mol) than that of another compound, viz., F0470-0003 and cocrystal, which showed binding affinity of −99.68 kcal/mol and −92.01 kcal/mol, respectively. Better efficacy of combined strategy of molecular docking and free energy calculations to predict the binding-free energy indicate that F1877-0839 may be used as lead discovery and optimization of macrodomain targeting SARS-CoV-2.

## 6. Catalytic Mechanism

In order to decipher the structural information on the binding sites, it is important to know the catalytic mechanism involved at the drug–protein interface. For “Compound ID: F1877-0839” residues, viz., Gly48, Val 49, Ala50, Ile131, Phe156, and Asp157, contributed via hydrogen bonding, while interacting residues, such as Val49, Leu126, and Phe156, interacted via hydrogen bonding in “Compound ID: F0470-0003”. We hypothesize it is the ribose scaffold of both compounds that induce the conformational change and compete with ADP-ribose binding site, paving the way for key interactions with Phe156 and disclosed potency inhibition of 79% for “Compound ID: F1877-0839” and 69% for “Compound ID: F0470-0003”. Moreover, differences in both core structures are due to the substitution of chlorobenzyl ring in “Compound ID: F0470-0003”, while substitution of oxygen at the ortho position, and this varying electron density might impact long-range electrostatic forces for receptor recognition. The peculiarity of our study identified water networks, which play an important role in protein–ligand recognition; however, their contribution to ligand binding is often difficult to identify. As shown in Figure S4C,D, we were able to identify the water networks. In continuation to such contribution’s residues, viz., Ala38, Lys44, Ala129, and Phe132, are involved in bridging interactions in “Compound ID: F1877-0839”. However, water-mediated interactions are also profound in “Compound ID: F0470-0003” with Ala38, Ala129, Ile131, and Phe132 residual contributions. Water networks reorganize upon ADPr binding, with a network of tightly bound water molecules acting as protein–ligand bridges; moreover, tightly bound water molecules are co-opted by fragment binding, with implications for inhibitor design. Ligand-induced conformational changes in the SARS-CoV-2 ADRP structure were prevalent in the simulated complex.

X-ray crystallography augments structure-based drug design and depends upon the accurate model of the shape, electrostatic potential, solvation and flexibility of the target site. Studies have found that the terminal ribose of ADPr adopts the β epimer, with the C1″ hydroxyl hydrogen bonded to the backbone nitrogen of Gly48. The proximal ribose ring is stabilized in the pocket by hydrophobic interactions with Phe132 and Ile131, as well as a set of hydrogen bonds with Gly46 (OH2, and Gly49 (OH1). All of these residues are conserved. The ligands and protein side chains are shown in ball-and-stick representation, with the ligand bonds coloured in purple. Hydrogen bonds are shown as green dotted lines, while the spoked arcs represent protein residues making nonbonded contacts with the ligand. The red circles and ellipses indicate protein residues (Refer Supplementary Figures S8–S10 that are in equivalent 3D positions when the two structural models are superposed and were generated using Ligplot^+^ [57]. Moreover, both contain the 1,3,4-thiadiazole ring at the central core region of the molecule and were suitably docked at the distal side of the binding pocket. Ligplot Interactions of docked Complex of Compound ID: F0470-0003 and schematic view of detailed ligand atom interactions with the protein residues is illustrated in Figures S8–S10 respectively.

The distal ribose ring is stabilized by the residues Pro125, Leu126 and Leu127 via hydrophobic interactions. The diphosphate moiety binds between two loops, that cover three segments with high sequence conservation, including a glycine-rich segment (Gly46-Gly47-Gly48) within the former loop; thus, it is conceivable that there is a conformational change in the binding pocket. Structural differences include the peptide flips of Gly48 and Ala129 that allow two new hydrogen bonds with the diphosphate portion of ADPr and a coupled conformational change in the Phe132 and Asn99 side chains accommodate the terminal ribose of ADPr. Residues Ala38, Gly48, Val49, Leu126, Ala129, Ile131, Phe132, and Phe156 contributed to hydrogen bonding interactions throughout the 200 ns simulation run. Screening of the top most inhibitors at the catalytic active site of macrodomain revealed that the protonation states of catalytic site residues and active site hydrogen bond networks in the docked complexes revealed the molecular basis of functionality relevant flexibility in the active site loops. The large number of bridging waters identified here provides an unmatched opportunity to systematically examine the thermodynamics of water displacement.

### Conformational Dynamics

RMSD of the protein Ca-backbone from its starting position increased to 1.4 Å for the first 100 ns and then became stable around 1.8 Å in the last 100 ns course of the simulation. Deviations converged and attained equilibrium during 75 ns simulation time of indicating 0.4 Å deviation in the protein–ligand (F1877-0839) complex, whereas in RMSD of the protein–ligand (F0470-0003) complex, it was quite different as we saw the complex had undergone a conformational change and simulations had not equilibrated much during the initial 50 ns simulation; however, with the simulation progression run, as represented in (Figure S3B), structural deviation with a magnitude of >1.8 Å was visible.

## 7. Conclusions

The present study illustrates molecular modelling insights into SARS-CoV-2 (WT) Spike inhibitors using experimental ACE2 Inhibition TR-FRET Assay, high throughput Screening and MDS. MDS results validated a corresponding experimental data of identified leads and found Compound ID: “F1877-0839” as the best drug among all, inhibiting Spike protein in micromolar range, thereby making it a therapeutic potential agent to treat Coronavirus. In parallel, this study is the first of its kind to identify novel pockets in SARS-CoV-2 dubbed FILLY pockets. Concurrently, this investigation represents a pioneering endeavor in the identification of previously uncharacterized binding sites in SARS-CoV-2 termed FILLY pockets. The potential involvement of FILLY pockets in mediating transport pathways and their putative relevance in the context of protein engineering necessitate further comprehensive exploration and investigation. Further, our studies are under process of determining how changing the functional group of the screened compounds identified through pseudovirus inhibition assay can impact the biological activity. Moreover, our findings could further be improved by exploring the other lead candidates by leveraging drug–target interactions along with experimentally known bindings by use of genetically expressed datasets, which, in addition to the clinical trial data outcomes, could be helpful for drug selection.

## 8. Significance

COVID-19 is triggered by infection with SARS-CoV-2 virus, wherein interaction between the receptor binding domain of the SARS-spike protein on surface of viral particle and its receptor present on cell surface of human cells, the angiotensin I, converts enzyme 2 (ACE2). Our findings suggest that electrostatic interactions are a major contributing factor for increased omicron transmissibility. Our structural modelling studies revealed that Spike receptor binding domain (S RBD) plays a pivotal role in enhancing ACE2 recognition. The unique contribution of our study lies in identification of water networks that play important role in protein–ligand recognition. Our molecular dynamics simulation results validated a corresponding experimental data of identified leads and found Compound ID: “F1877-0839” as the best drug, inhibiting Spike protein in micromolar range, thereby making it a therapeutic potential agent to treat coronavirus. Moreover, this study is the first of the kind to identify the novel FILLY pockets in SARS-CoV-2.

## 9. Limitations

With the unprecedented number of deaths as the experts forecast for the next several years and the recent emergence of XBB recombinant variants, the mechanisms of SARS-CoV-2 entry into host cells still remain a mystery, and could be further explored by focusing on different targets, for which our studies are under process. Greater focus on exploring other receptors, viz., Kidney Injury Molecule-1/T cell immunoglobulin mucin domain 1 (KIM-1/TIM-1), tyrosine-protein kinase receptor UFO (AXL), lectins, Cathepsins, etc., will further help in exploring their importance, which might help in deciphering their important role in promoting viral infection of the human respiratory system and indicate their role as alternative receptors for future clinical intervention strategies.

## Figures and Tables

**Table 1:** Effects of Compounds on WT Spike: ACE2 Binding: Inhibitory effects of compounds against Spike: ACE2 binding are summarized in Table 1.

Inhibitor, Conc.	Percent Inhibition
F5084-0852, 30 µM	0
F1877-0839, 30 µM	79
F2619-0022, 30 µM	30
F1877-1292, 30 µM	12
F0466-0005, 30 µM	0
F2085-0027, 30 µM	2
F0772-2453, 30 µM	43
F0470-0003, 30 µM	69
F0827-0193, 30 µM	38
F2173-1125, 30 µM	0
Anti-Spike, 0.0001 µM	21
Anti-Spike, 0.001 µM	49
Anti-Spike, 0.01 µM	76

**Table 2:** Data for the Effect of Compounds 1–10 on WT Spike: ACE2 Binding.

Compound I.D.	TR-FRET Ratio		% Activity		% Inhibition
	Repeat 1	Repeat 2	Repeat 1	Repeat 2	
No Compound	0.99	1.00	99	101	0
F5084-0852, 30 µM	1.00	1.03	103	111	0
F1877-0839, 30 µM	0.77	0.77	20	21	79
F2619-0022, 30 µM	0.89	0.93	62	77	30
F1877-1292, 30 µM	0.96	0.96	88	89	12
F0466-0005, 30 µM	1.01	0.99	104	97	0
F2085-0027, 30 µM	0.97	1.00	93	103	2
F0772-2453, 30 µM	0.89	0.86	62	51	43
F0470-0003, 30 µM	0.80	0.79	32	29	69
F0827-0193, 30 µM	0.89	0.89	63	61	38
F2173-1125, 30 µM *	1.15	1.20	153	172	0
Anti-Spike, 0.0001 µM	0.92	0.95	72	86	21
Anti-Spike, 0.001 µM	0.87	0.85	54	49	49
Anti-Spike, 0.01 µM	0.79	0.77	27	22	76
Background	0.71	0.71			

* Compound was brightly coloured in the assay, leading to interference with the TR-FRET signal in assay.

**Table 3:** Free energy calculations (MM-GBSA) of identified lead compounds and Cocrystal.

Name of Compound	Solv GB	vdW	Coulomb	Covalent	Hbond	∆G_Total_ (kcal/mol)
F1877-0839	29.66 ± 6.24	−72.14 ± 2.96	−233.35 ± 2.03	14.97 ± 0.02	−0.86 ± 0.14	−**106.38** ± 1.56
F0470-0003	48.16 ± 5.16	−54.17 ± 2.89	−40.41 ± 2.46	1.23 ± 0.30	−0.78 ± 0.11	−**99.68** ± 1.52
Cocrystal	23.75 ± 5.09	−62.55 ± 2.78	−31.65 ±2.17	6.7 ± 0.27	−7.78 ± 0.10	−92.01 ± 1.58

The bold values represent the best among the given factors.

## Data Availability

Not applicable.

## Supplementary Materials

The following supporting information can be downloaded at: https://www.mdpi.com/article/10.3390/bioengineering10080961/s1, Figure S1 (A): Serial dilutions of 10 patient serum samples were individually mixed with the pseudotyped viruses at 37 C for 1 h before being added to Huh-7 cells for incubation of 24 h to determine the EC50; Figure S1 (B): Human CoVs bind to ADP-ribose with similar affinity where (A and B) depict the binding of human Mdo2 and SARS-CoV, MERS-CoV & SARS-CoV-2 Mac1 proteins; Figure S2: Representative Figure, The SARS-CoV-2 conserved macrodomain is a mono-ADP-ribosylhydrolase, Reprinted with permission from Ref. [23]; Figure S3: Top most selected ligands used for checking the efficacy of Effects of Compounds on WT Spike: ACE2 Binding; Figure S4: (A&B) Ligand properties in terms of PSA, SASA, and rGYR illustrating stability of simulated docked complexes of F1877-0839 and F0470-0003 respectively. (C&D): Simulated Interaction diagram of F1877-0839 and F0470-0003; Figure S5: (A&B) Torsional profile of the drugs; F1877-0839 and F0470-0003. (C &D): correspond to the respective RMSF fluctuation of the drugs at the active site of the ligand; Figure S6: Docked complex of Compound ID: F1877-0839 WT Spike: ACE2 Binding (A) Labelled with Interacting residues (B) without interacting residues; Figure S7: Docked complex of Compound ID: F0470-0003 WT Spike: ACE2 Binding (A) Labelled with Interacting residues (B) without interacting residues; Figure S8: (Left): Ligplot Interactions of docked Complex of Compound ID: F1877-0839. (Right): schematic view of detailed ligand atom interactions with the protein residue; Figure S9: (Left): Ligplot Interactions of docked Complex of Compound ID: F0470-0003. (Right): schematic view of detailed ligand atom interactions with the protein residue; Figure S10: (Left): Ligplot Interactions of cocrystal Docked Complex. (Right): schematic view of detailed ligand atom interactions with the protein residue; Table S1: Convolutional deep neural network-based approach named DOcking decoy selection with Voxel-based deep neural nEtwork (DOVE) for evaluating protein docking for SARS-CoV-2 models; Table S2: Enzymes and Substrates used for experiment; Table S3: List of Compounds and their test range used in this study for WT Spike: ACE2 Binding; Table S4: Induced fit Docking results of all the selected compounds and cocrystal at the active site of the macrodomain; Table S5: ADMET analysis of the inhibitors used in this study.

## Abbreviations

MDS	Molecular Dynamics Simulations
ACE2	Angiotensin-Converting Enzyme 2
TR-FRET	Time Resolved Forster/Fluorescence energy transfer
HTVS	High-Throughput Virtual Screening
XP	Extra Precision
QPLD	Quantum Polarised Ligand Docking

## References

[1] Suzuki R., Yamasoba D., Kimura I., Wang L., Kishimoto M., Ito J., Morioka Y., Nao N., Nasser H., Uriu K. (2022). Attenuated fusogenicity and pathogenicity of SARS-CoV-2 Omicron variant. Nature.

[2] WHO Coronavirus (COVID-19) Dashboard. https://covid19.who.int/.

[3] Lv M., Luo X., Estill J., Liu Y., Ren M., Wang J., Wang Q., Zhao S., Wang X., Yang S. (2020). On Behalf of the COVID-Evidence and Recommendations Working Group. Coronavirus disease (COVID-19): A scoping review. Eurosurveillance.

[4] Yang X., Yu Y., Xu J., Shu H., Xia J., Liu H., Wu Y., Zhang L., Yu Z., Fang M. (2020). Clinical course and outcomes of critically ill patients with SARS-CoV-2 pneumonia in Wuhan, China: A single-centered, retrospective, observational study. Lancet Respir. Med..

[5] Yoshimoto F.K. (2020). The Proteins of Severe Acute Respiratory Syndrome Coronavirus-2 (SARS CoV-2 or n-COV19), the Cause of COVID-19. Protein J..

[6] Finkel Y., Mizrahi O., Nachshon A., Weingarten-Gabbay S., Morgenstern D., Yahalom-Ronen Y., Tamir H., Achdout H., Stein D., Israeli O. (2020). The coding capacity of SARS-CoV-2. Nature.

[7] Chan J.F.-W., Kok K.-H., Zhu Z., Chu H., To K.K.-W., Yuan S., Yuen K.-Y. (2020). Genomic characterization of the 2019 novel human-pathogenic coronavirus isolated from a patient with atypical pneumonia after visiting Wuhan. Emerg. Microbes Infect..

[8] Cecon E., Burridge M., Cao L., Carter L., Ravichandran R., Dam J., Jockers R. (2021). SARS-CoV-2 spike binding to ACE2 in living cells monitored by TR-FRET. Cell Chem. Biol..

[9] Lan J., Ge J., Yu J., Shan S., Zhou H., Fan S., Zhang Q., Shi X., Wang Q., Zhang L. (2020). Structure of the SARS-CoV-2 spike receptor-binding domain bound to the ACE2 receptor. Nature.

[10] Letko M., Marzi A., Munster V. (2020). Functional assessment of cell en- try and receptor usage for SARS-CoV-2 and other lineage B betacoronaviruses. Nat. Microbiol..

[11] Hulswit R.J.G., De Haan C.A.M., Bosch B.J. (2016). Coronavirus Spike Protein and Tropism Changes. Adv. Virus Res..

[12] Carabelli A.M., Peacock T.P., Thorne L.G., Harvey W.T., Hughes J., Peacock S.J., Barclay W.S., de Silva T.I., Towers G.J., COVID-19 Genomics UK Consortium (2023). SARS-CoV-2 variant biology: Immune escape, transmission and fitness. Nat. Rev. Microbiol..

[13] Tamura T., Ito J., Uriu K., Zahradnik J., Kida I., Anraku Y., Nasser H., Shofa M., Oda Y., Lytras S. (2023). Virological characteristics of the SARS-CoV-2 XBB variant derived from recombination of two Omicron subvariants. Nat. Commun..

[14] Li J., Lai S., Gao G.F., Shi W. (2021). The emergence, genomic diversity and global spread of SARS-CoV-2. Nature.

[15] Yurkovetskiy L., Wang X., Pascal K.E., Tomkins-Tinch C., Nyalile T.P., Wang Y., Baum A., Diehl W.E., Dauphin A., Carbone C. (2020). Structural and functional analysis of the D614G SARS-CoV-2 spike protein variant. Cell.

[16] Hadfield J., Megill C., Bell S.M., Huddleston J., Potter B., Callender C., Sagulenko P., Bedford T., Neher R.A. (2018). Nextstrain: Real-time tracking of pathogen evolution. Bioinformatics.

[17] Imbert I., Snijder E.J., Dimitrova M., Guillemot J.-C., Lécine P., Canard B. (2008). The SARS-Coronavirus PLnc domain of nsp3 as a replication/transcription scaffolding protein. Virus Res..

[18] Shan H., Liu J., Shen J., Dai J., Xu G., Lu K., Han C., Wang Y., Xu X., Tong Y. (2021). Development of potent and selective inhibitors targeting the papain-like protease of SARS-CoV-2. Cell Chem. Biol..

[19] Sastry G.M., Adzhigirey M., Day T., Annabhimoju R., Sherman W. (2013). Protein and ligand preparation: Parameters, protocols, and influence on virtual screening enrichments. J. Comput. Aided Mol. Des..

[20] Guo Y.R., Cao Q.D., Hong Z.S., Chen S.D., Jin H.J., Tan K.S., Wang D.Y., Yan Y. (2020). The origin, transmission and clinical therapies on coronavirus disease 2019 (COVID-19) outbreak—An update on the status. Mil. Med. Res..

[21] Wu A., Peng Y., Huang B., Ding X., Wang X., Niu P., Meng J., Zhu Z., Zhang Z., Wang J. (2020). Genome Composition and Divergence of the Novel Coronavirus (2019-nCoV) Originating in China. Cell Host Microbe.

[22] Srinivasan S., Cui H., Gao Z., Liu M., Lu S., Mkandawire W., Narykov O., Sun M., Korkin D. (2020). Structural genomics of SARS-CoV-2 indicates evolutionary conserved functional regions of viral proteins. Viruses.

[23] Alhammad Y.M.O., Kashipathy M.M., Roy A., Gagné J.-P., McDonald P., Gao P., Nonfoux L., Battaile K.P., Johnson D.K., Holmstrom E.D. (2021). The SARS-CoV-2 Conserved Macrodomain Is a Mono-ADP-Ribosylhydrolase. J. Virol..

[24] Egloff M.-P., Malet H., Putics A., Heinonen M., Dutartre H., Frangeul A., Gruez A., Campanacci V., Cambillau C., Ziebuhr J. (2006). Structural and Functional Basis for ADP-Ribose and Poly(ADP-Ribose) Binding by Viral Macro Domains. J. Virol..

[25] Putics A., Gorbalenya A.E., Ziebuhr J. (2006). Identification of protease and ADP-ribose 1’’-monophosphatase activities associated with transmissible gastroenteritis virus non-structural protein 3. J. Gen. Virol..

[26] Saikatendu K.S., Joseph J.S., Subramanian V., Clayton T., Griffith M., Moy K., Velasquez J., Neuman B.W., Buchmeier M.J., Stevens R.C. (2005). Structural Basis of Severe Acute Respiratory Syndrome Coronavirus ADP-Ribose-1″-Phosphate Dephosphorylation by a Conserved Domain of nsP3. Structure.

[27] Cho C.C., Lin M.H., Chuang C.Y., Hsu C.H. (2016). Macro domain from Middle East respiratory syndrome coronavirus (MERS-CoV) is an efficient ADP-ribose binding module: Crystal structure and biochemical studies. J. Biol. Chem..

[28] Xu Y., Cong L., Chen C., Wei L., Zhao Q., Xu X., Ma Y., Bartlam M., Rao Z. (2009). Crystal Structures of Two Coronavirus ADP-Ribose-1″-Monophosphatases and Their Complexes with ADP-Ribose: A Systematic Structural Analysis of the Viral ADRP Domain. J. Virol..

[29] Rack J.G.M., Perina D., Ahel I. (2016). Macrodomains: Structure, Function, Evolution, and Catalytic Activities. Annu. Rev. Biochem..

[30] Neuman B.W. (2016). Bioinformatics and functional analyses of coronavirus nonstructural proteins involved in the formation of replicative organelles. Antivir. Res..

[31] Lei J., Kusov Y., Hilgenfeld R. (2017). Nsp3 of coronaviruses: Structures and functions of a large multi-domain protein. Antivir. Res..

[32] Perina D., Mikoč A., Ahel J., Ćetković H., Žaja R., Ahel I. (2014). Distribution of protein poly(ADPribosyl)ation systems across all domains of life. DNA Repair..

[33] Claverie J.-M. (2020). A Putative Role of de-Mono-ADP-Ribosylation of STAT1 by the SARS-CoV-2 Nsp3 Protein in the Cytokine Storm Syndrome of COVID-19. Viruses.

[34] Dhankhar P., Dalal V., Kumar V. (2021). Screening of Severe Acute Respiratory Syndrome Coronavirus 2 RNA-Dependent RNA Polymerase Inhibitors Using Computational Approach. J. Comput. Biol..

[35] Ghufran M., Ullah M., Khan H.A., Ghufran S., Ayaz M., Siddiq M., Abbas S.Q., Hassan S.S.U., Bungau S. (2023). In-Silico Lead Druggable Compounds Identification against SARS COVID-19 Main Protease Target from In-House, Chembridge and Zinc Databases by Structure-Based Virtual Screening, Molecular Docking and Molecular Dynamics Simulations. Bioengineering.

[36] Wang X., Terashi G., Christoffer C.W., Zhu M., Kihara D. (2019). Protein docking model evaluation by 3D deep convolutional neural networks. Bioinformatics.

[37] Laurini E., Marson D., Aulic S., Fermeglia M., Pricl S. (2020). Computational Alanine Scanning and Structural Analysis of the SARS-CoV-2 Spike Protein/Angiotensin-Converting Enzyme 2 Complex. ACS Nano.

[38] Han P., Li L., Liu S., Wang Q., Zhang D., Xu Z., Han P., Li X., Peng Q., Su C. (2022). Receptor binding and complex structures of human ACE2 to spike RBD from omicron and delta SARS-CoV-2. Cell.

[39] Willett B.J., Grove J., MacLean O.A., Wilkie C., De Lorenzo G., Furnon W., Cantoni D., Scott S., Logan N., Ashraf S. (2022). SARS-CoV-2 Omicron is an immune escape variant with an altered cell entry pathway. Nat. Microbiol..

[40] Li Q., Wu J., Nie J., Zhang L., Hao H., Liu S., Zhao C., Zhang Q., Liu H., Nie L. (2020). The Impact of Mutations in SARS-CoV-2 Spike on Viral Infectivity and Antigenicity. Cell.

[41] Fehr A.R., Channappanavar R., Jankevicius G., Fett C., Zhao J., Athmer J., Meyerholz D.K., Ahel I., Perlman S. (2016). The Conserved Coronavirus Macrodomain Promotes Virulence and Suppresses the Innate Immune Response during Severe Acute Respiratory Syndrome Coronavirus Infection. mBio.

[42] Eriksson K.K., Cervantes-Barragán L., Ludewig B., Thiel V. (2008). Mouse hepatitis virus liver pathology is dependent on ADP-ribose-1″-phosphatase, a viral function conserved in the alpha-like supergroup. J. Virol..

[43] Putics A., Filipowicz W., Hall J., Gorbalenya A.E., Ziebuhr J. (2005). ADP-Ribose-1″-Monophosphatase: A Conserved Coronavirus Enzyme That Is Dispensable for Viral Replication in Tissue Culture. J. Virol..

[44] Fehr A.R., Athmer J., Channappanavar R., Phillips J.M., Meyerholz D.K., Perlman S. (2014). The nsp3 Macrodomain Promotes Virulence in Mice with Coronavirus-Induced Encephalitis. J. Virol..

[45] Fehr A.R., Singh S.A., Kerr C.M., Mukai S., Higashi H., Aikawa M. (2020). The impact of PARPs and ADP-ribosylation on inflammation and host–pathogen interactions. Genes Dev..

[46] Jorgensen W.L., Chandrasekhar J., Madura J.D., Impey R.W., Klein M.L. (1983). Comparison of simple potential functions for simulating liquid water. J. Chem. Phys..

[47] Bowers K.J., Chow D.E., Xu H., Dror R.O., Eastwood M.P., Gregersen B.A., Klepeis J.L., Kolossvary I., Moraes M.A., Sacerdoti F.D. Scalable Algorithms for Molecular Dynamics Simulations on Commodity Clusters. Proceedings of the SC’06: 2006 ACM/IEEE Conference on Supercomputing.

[48] Iqbal S., Potharaju R., Naveen S., Lokanath N.K., Mohanakrishnan A.K., Gunasekaran K. (2021). Design, crystal structure determination, molecular dynamic simulation and MMGBSA calculations of novel p38-alpha MAPK inhibitors for combating Alzheimer’s disease. J. Biomol. Struct. Dyn..

[49] Case D.A., Darden T., Cheatham T.E., Simmerling C., Wang J., RDuke E., Luo R., Crowley M., Walker R., Zhang W. (2008). Amber. Constant Press. Mol. Dyn. Algorithms.

[50] Darden T., York D., Pedersen L. (1993). Particle mesh Ewald: An N log(N) method for Ewald sums in large systems. J. Chem. Phys..

[51] Miller B.R., McGee T.D., Swails J.M., Homeyer N., Gohlke H., Roitberg A.E. (2012). *MMPBSA.py*: An Efficient Program for End-State Free Energy Calculations. J. Chem. Theory Comput..

[52] Kumari R., Kumar V., Dhankhar P., Dalal V. (2022). Promising antivirals for PLpro of SARS-CoV-2 using virtual screening, molecular docking, dynamics, and MMPBSA. J. Biomol. Struct. Dyn..

[53] Kalathiya U., Padariya M., Mayordomo M., Lisowska M., Nicholson J., Singh A., Baginski M., Fahraeus R., Carragher N., Ball K. (2020). Highly conserved homotrimer cavity formed by the SARS-CoV-2 spike glycoprotein: A novel binding site. J. Clin. Med..

[54] Weiner P.K., Langridge R., Blaney J.M., Schaefer R., Kollman P.A. (1982). Electrostatic potential molecular surfaces. Proc. Natl. Acad. Sci. USA.

[55] McCoy A.J., Epa V.C., Colman P.M. (1997). Electrostatic complementarity at protein/protein interfaces. J. Mol. Biol..

[56] Gan H.H., Zinno J., Piano F., Gunsalus K.C. (2022). Omicron Spike Protein Has a Positive Electrostatic Surface That Promotes ACE2 Recognition and Antibody Escape. Front. Virol..

[57] Laskowski R.A., Swindells M.B. (2011). LigPlot+: Multiple ligand–protein interaction diagrams for drug discovery. J. Chem. Inf. Model..

