# Supplementary material for: Deep Drug Discovery of Mac Domain of SARS-CoV-2 (WT) Spike Inhibitors: Using Experimental ACE2 Inhibition TR-FRET Assay, Screening, Molecular Dynamic Simulations and Free Energy Calculations
Source: Bioengineering (Basel). 2023 Aug 14;10(8):961. doi: 10.3390/bioengineering10080961 (PMC10451221; doi:10.3390/bioengineering10080961)
Supplement: Supplementary file 1 [file bioengineering-10-00961-s001.zip › bioengineering-2440434-supplementary.pdf]

bioengineering

Supporting Information

Deep Drug Discovery of Mac Domain of SARS-CoV-2 (WT) Spike Inhibitors: Using Experimental ACE2 Inhibition TR-FRET Assay, Screening, Molecular Dynamic Simulations and Free Energy Calculations

Saleem Iqbal * and Sheng-Xiang Lin *

Axe Molecular Endocrinology and Nephrology, CHU Research Center and Laval University, Quebec City, QC G1V 4G2, Canada
* Correspondence: Saleem.iqbal.1@ulaval.ca (S.I.); sheng-xiang.lin@crchudequebec.ulaval.ca (S.-X.L.)

Supplementary Data

Table S1. Convolutional deep neural network-based approach named DOcking decoy selection with Voxel-based deep neural nEtwork (DOVE) for evaluating protein docking for SARS-CoV-2 models.

PDB entry	ATOM+GOAP+ITScore*
6WOJ	0.49124
6M0J	0.17618

* Input based on atom types, locations, and atoms' GOAP and ITScore energy scores in a 403 A^3 cube in the interface area(within 10A), in which the cube center is the interface area center.

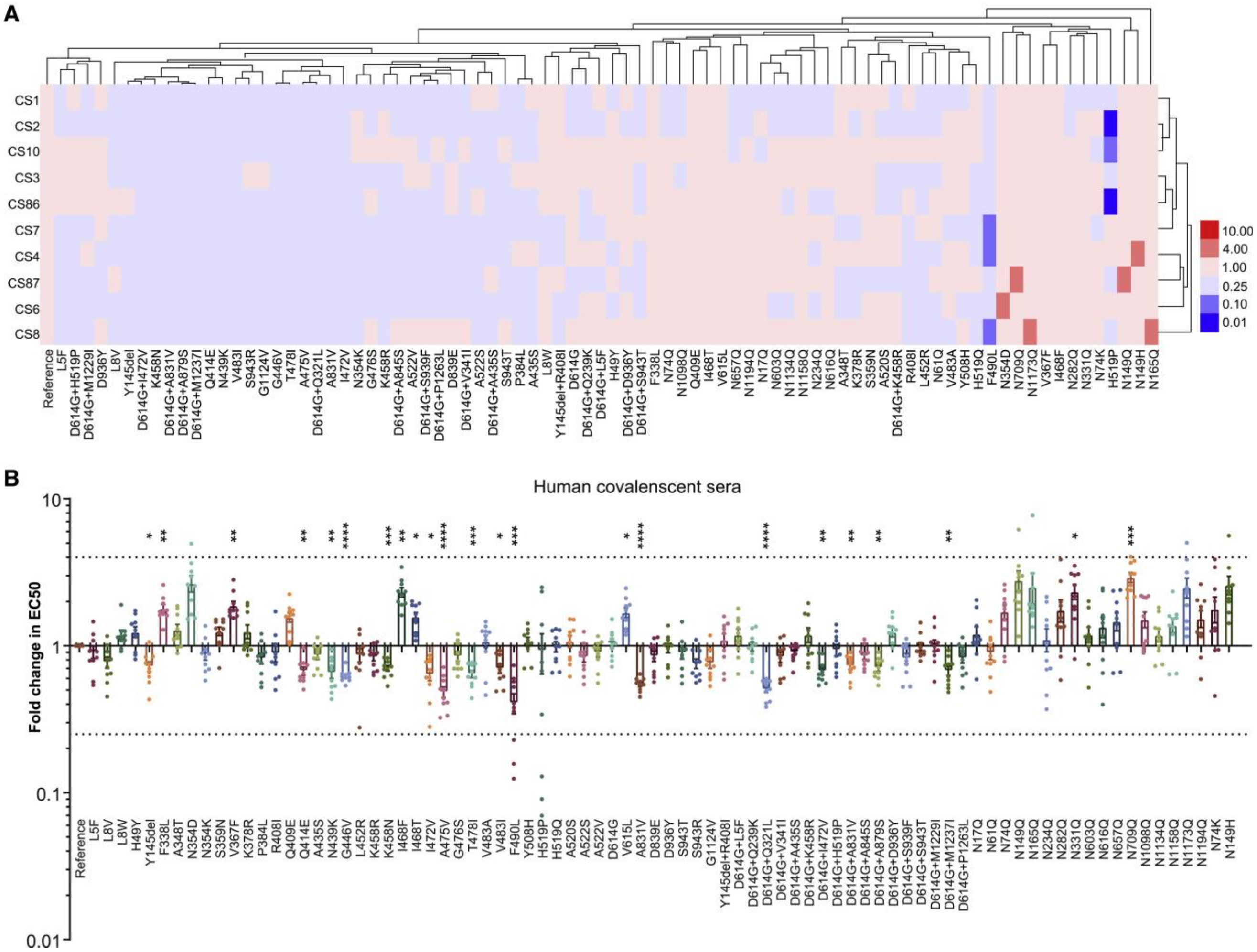

Figure S1. (A). (A) Serial dilutions of 10 patient serum samples were individually mixed with the pseudotyped viruses at 37 C for 1 h before being added to Huh-7 cells for incubation of 24 h to determine the EC50. The experiments were repeated at least 3 times. Hem I software was used to analyzing the data and draw the heatmap. The red and blue boxes indicate the increase or decrease of the neutralization activity as shown in the scale bar. (B) Summary of the data from ten serum samples, with the values presented as mean ± SEM. The horizontal dashed lines indicate the threshold of 4-fold difference. *p < 0.05, **p < 0.01, ***p < 0.005, ****p < 0.001. **Figure S1 (B)**. Human CoVs bind to ADP-ribose with similar affinity where (A and B) depict the binding of human Mdo2 and SARS-CoV, MERS-CoV & SARS-CoV-2 Mac1 proteins.

A

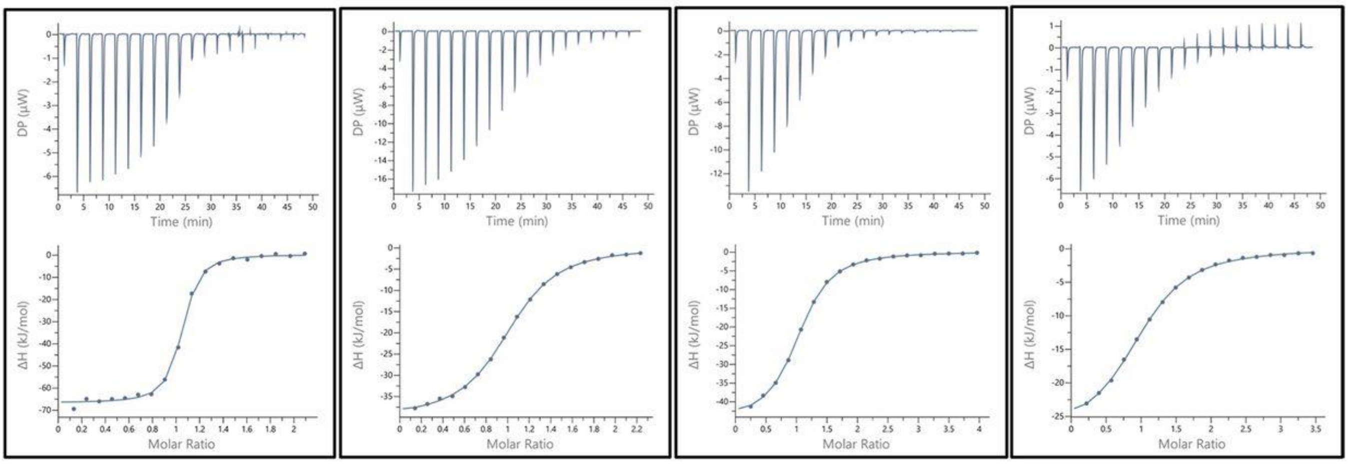

B

Macrodomain	Stoichiometry (N)	Kd (μM)	ΔH (kcal/mol)	ΔG (kcal/mol)
Mdo2	0.92 ± 0.01	0.24 ± 0.02	-66 ± 1	-38 ± 2
SARS-CoV	0.89 ± 0.04	10.8 ± 1.7	-40 ± 1.2	-28 ± 0.4
MERS-CoV	0.97 ± 0.04	7.9 ± 0.15	-47 ± 3	-29 ± 0.4
SARS-CoV-2	1.14 ± 0.06	16.8 ± 0.04	-28 ± 0.1	-27 ± 0.1

C

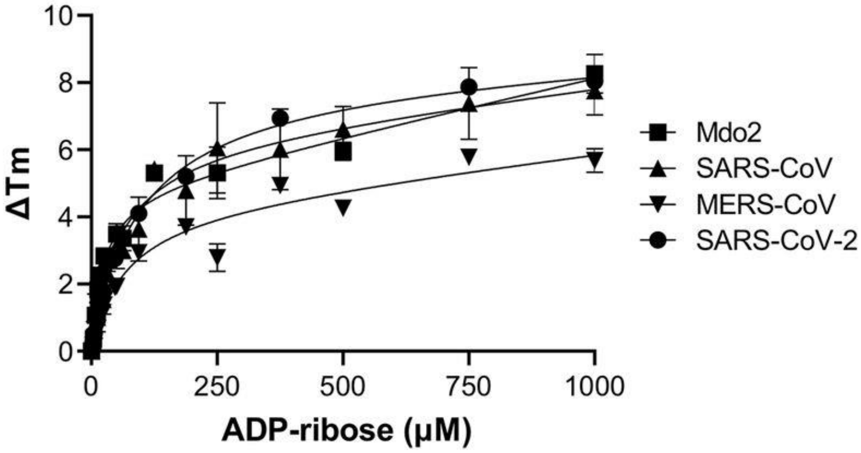

Figure S2. (A&B) All CoV Mac1 proteins bound to ADP-ribose with low micromolar affinity (7 to 16mM), while human Mdo2 bound with an affinity at least 30 times stronger (220 nM). Figure S2 (C). All four macrodomains subjected to assessment displayed elevated denaturation temperatures upon the introduction of ADP-ribose evidenced through thermal shift Assay**.** Representative Figure., Alhammad YMO, Kashipathy MM, Roy A, Gagné J-P, McDonald P, Gao P, Nonfoux L, Battaile KP, Johnson DK, Holmstrom ED, Poirier GG, Lovell S, Fehr AR. 2021. The SARS-CoV-2 conserved macrodomain is a mono-ADP-ribosylhydrolase. Reprinted with permission from Ref.[23].

Table S2. Enzymes and Substrates used for experiment.

Enzyme	Catalog #	Protein lot #	Assay concentration
ACE2-Eu	100705	210701	5 ng/rxn
WT Spike S1-biotin	100720	200423	100 ng/rxn

Table S3. List of Compounds and their test range used in this study for WT Spike: ACE2 Binding.

Compound I.D.	Compound Supplied	Stock Concentration	Dissolving Solvent	Test Range (μM)	Intermediate Dilution
F5084-0852	Solid	10 mM	DMSO	30	4 % DMSO in Assay Buffer
F1877-0839	Solid	10 mM	DMSO	30	4 % DMSO in Assay Buffer
F2619-0022	Solid	10 mM	DMSO	30	4 % DMSO in Assay Buffer
F1877-1292	Solid	10 mM	DMSO	30	4 % DMSO in Assay Buffer
F0466-0005	Solid	10 mM	DMSO	30	4 % DMSO in Assay Buffer
F2085-0027	Solid	10 mM	DMSO	30	4 % DMSO in Assay Buffer
F0772-2453	Solid	10 mM	DMSO	30	4 % DMSO in Assay Buffer
F0470-0003	Solid	10 mM	DMSO	30	4 % DMSO in Assay Buffer
F0827-0193	Solid	10 mM	DMSO	30	4 % DMSO in Assay Buffer
F2173-1125	Solid	10 mM	DMSO	30	4 % DMSO in Assay Buffer
Anti-Spike*	Solution	9 μM	PBS	0.0001, 0.001, 0.01	Assay Buffer

* Reference inhibitor

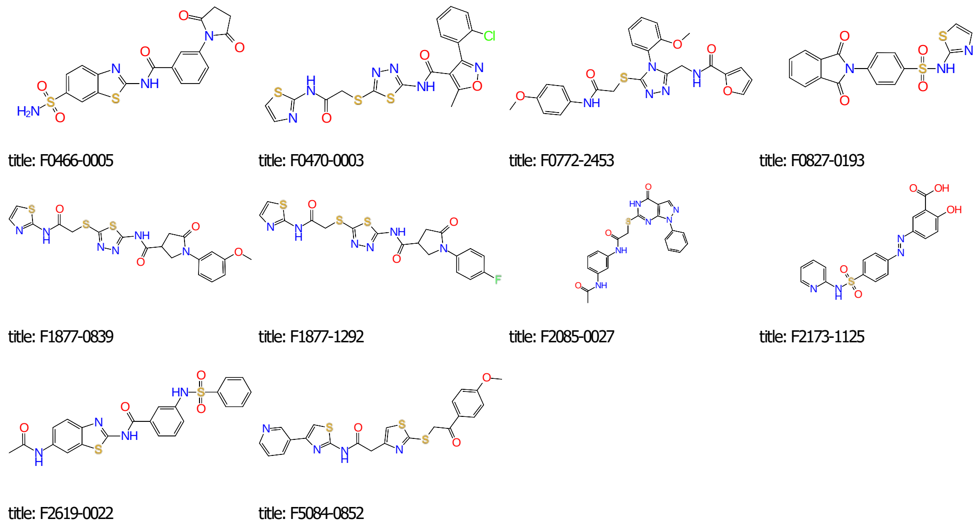

Figure S3. Top most selected ligands used for checking the efficacy of Effects of Compounds on WT Spike: ACE2 Binding.

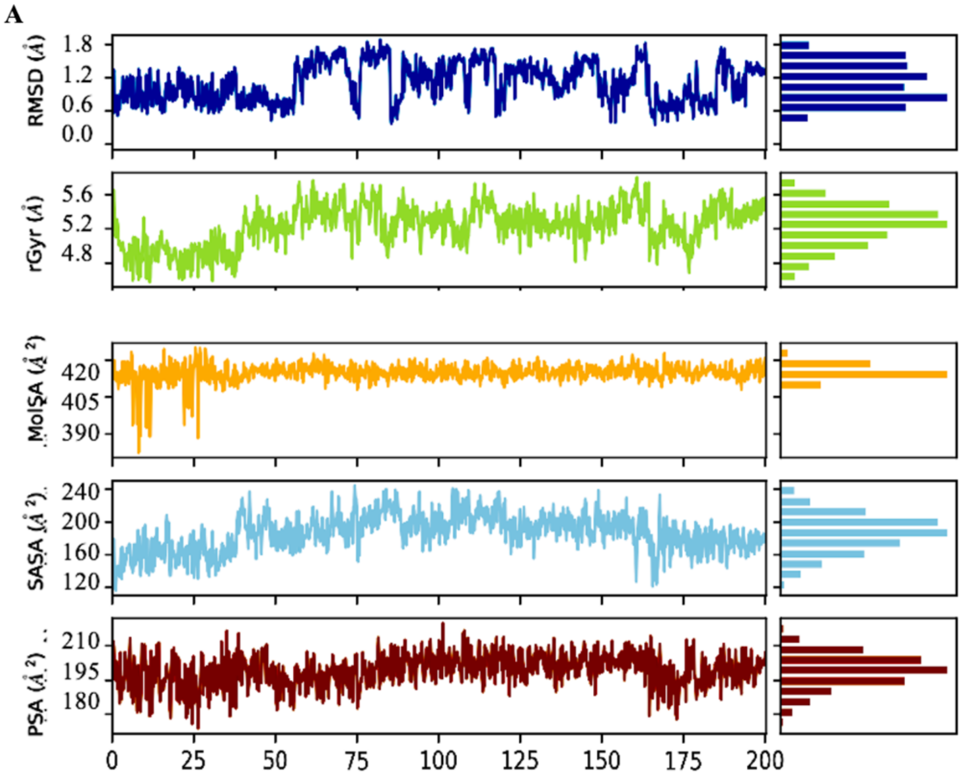

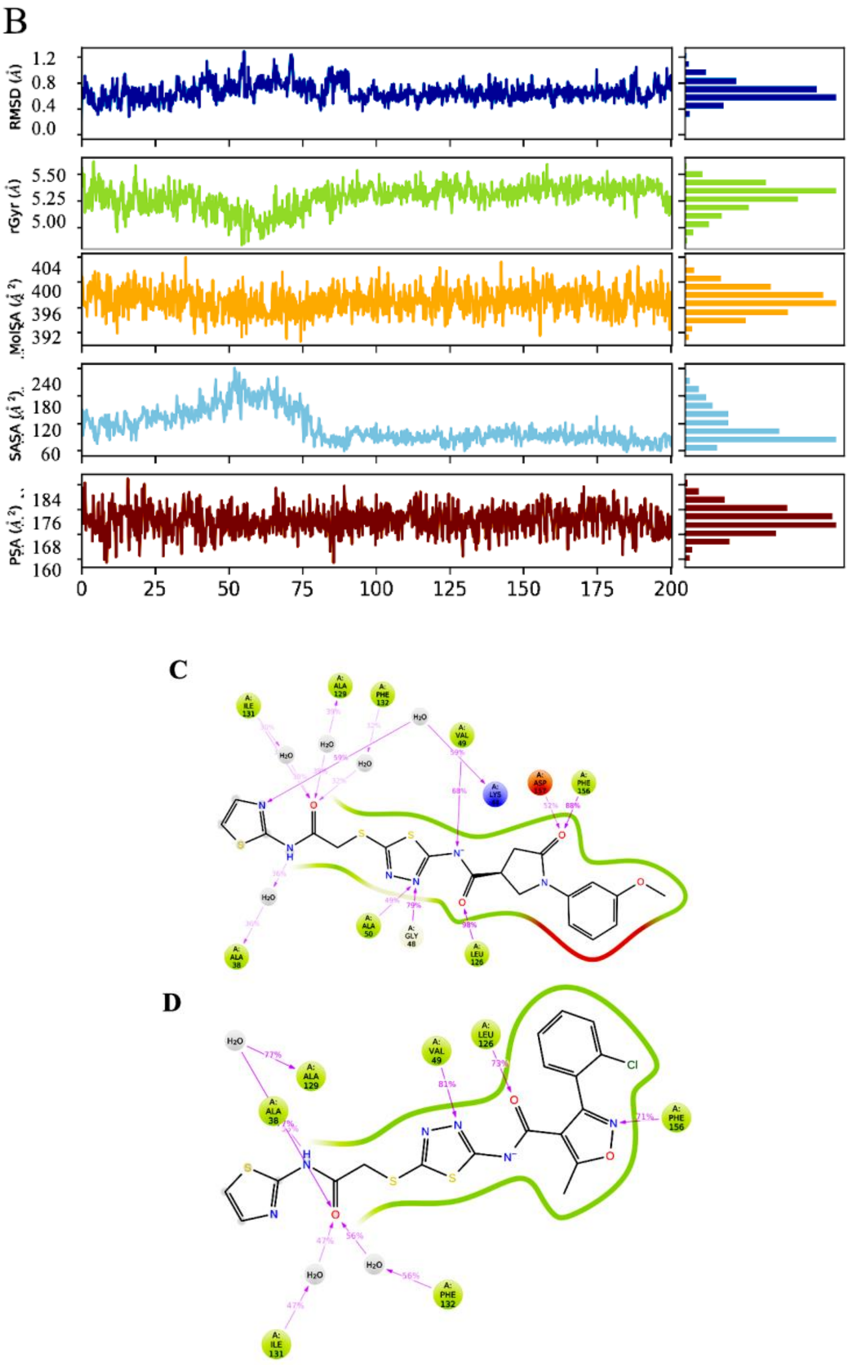

Figure S4. (A&B) Ligand properties in terms of PSA, SASA, and rGYR illustrating stability of simulated docked complexes of F1877-0839 and F0470-0003 respectively. (C&D): Simulated Interaction diagram of F1877-0839 and F0470-0003.

All the compounds for HTVS shown in **Figure S4**. were prepared and energy minimized using the Ligprep module of the Schrodinger (Schrodinger 14-2; Sastry, Adzhigirey, Day, Annabhi- moju, & Sherman, 2013), where (with) probable tautomeric and ionization states at pH = 7 ± 1 followed by minimization with OPLS 2005 force field. The ligands shown in Figure S3 were evaluated for checking the efficacy of Effects of Compounds on WT Spike: ACE2 Binding.

Table S4. Induced fit Docking results of all the selected compounds and cocrystal at the active site of the macrodomain.

Compound ID	Docking Score	Glide Energy kcal/mol
F5084-0852	-9.12	-59.35
F1877-0839	-12.87	-78.24
F2619-0022	-8.25	-61.34
F1877-1292	-8.68	-52.48
F0466-0005	-9.01	-60.62
F2085-0027	-10.32	-68.03
F0772-2453	-11.69	-70.04
F0470-0003	-9.61	-75.64
F0827-0193	-9.08	-54.86
F2173-1125	-8.88	-59.42
Cocrystal	-11.19	-75.02

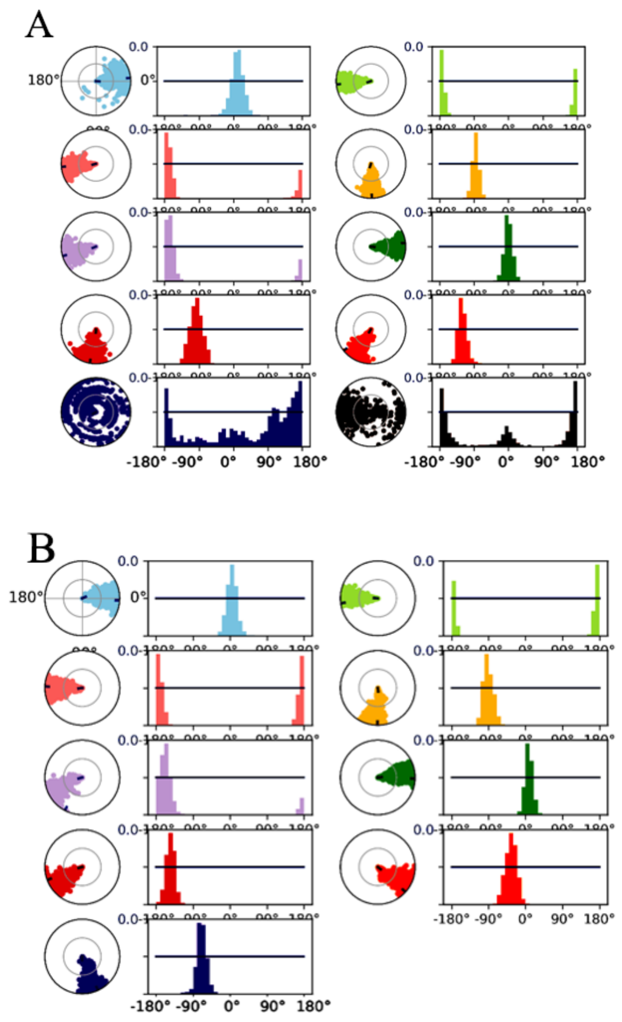

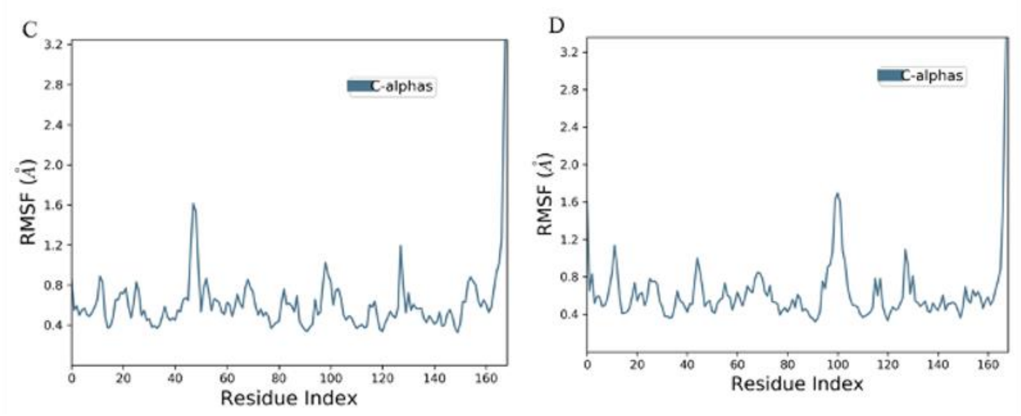

Figure S5. (A&B) Torsional profile of the drugs; F1877-0839 and F0470-0003. (C &D): correspond to the respective RMSF fluctuation of the drugs at the active site of the ligand.

Table S5. ADMET analysis of the inhibitors used in this study.

Title	FORMULA	MOL_WEIGHT	NAME	donorHB	accptHB	QPlogPoct	QPlogPw	QPlogPo/w	QPlogS	QPlogBB	QPlogKp	HumanOralAbsorption	PercentHumanOralAbsorption	RuleOfFive	RuleOfThree
F0466-0005	C18H14N4O5S2	430.46	3-(2,5-dioxopyrrolidin-1-yl)-N-(6-sulfamoyl-1,3-benzothiazol-2-yl)benzamide	3	11.5	26.468	19.954	0.352	-4.669	-2.793	-5.758	2	49.999	0	1
F0470-0003	C18H13ClN6O3S3	492.98	3-(2-chlorophenyl)-5-methyl-N-[5-({[(1,3-thiazol-2-yl)carbamoyl]methyl}sulfanyl)-1,3,4-thiadiazol-2-yl]-1,2-oxazole-4-carboxamide	2	10	25.24	16.359	3.262	-6.744	-1.492	-3.336	1	86.156	0	1
F0772-2453	C24H23N5O5S	493.53	N-{[4-(2-methoxyphenyl)-5-({[(4-methoxyphenyl)carbamoyl]methyl}sulfanyl)-4H-1,2,4-triazol-3-yl]methyl}furan-2-carboxamide	2	9	24.336	14.841	4.153	-5.528	-1.074	-1.327	3	100	0	0
F0827-0193	C17H11N3O4S2	385.42	4-(1,3-dioxo-2,3-dihydro-1H-isoindol-2-yl)-N-(1,3-thiazol-2-yl)benzene-1-sulfonamide	0	7	17.522	11.43	2.399	-4.276	-1.347	-3.216	3	81.402	0	0
F1877-0839	C19H18N6O4S3	490.58	1-(3-methoxyphenyl)-5-oxo-N-[5-({[(1,3-thiazol-2-yl)carbamoyl]methyl}sulfanyl)-1,3,4-thiadiazol-2-yl]pyrrolidine-3-carboxamide	2	12.25	27.589	18.377	2.323	-6.227	-2.026	-3.739	1	77.017	0	1
F1877-1292	C18H15FN6O3S3	478.54	1-(4-fluorophenyl)-5-oxo-N-[5-({[(1,3-thiazol-2-yl)carbamoyl]methyl}sulfanyl)-1,3,4-thiadiazol-2-yl]pyrrolidine-3-carboxamide	2	11.5	27.025	17.863	2.502	-6.409	-1.753	-3.669	1	79.136	0	1
F2085-0027	C21H18N6O3S	434.47	N-(3-acetamidophenyl)-2-({4-oxo-1-phenyl-1H,4H,5H-pyrazolo[3,4-d]pyrimidin-6-yl}sulfanyl)acetamide	3	9.5	26.614	18.355	2.483	-5.973	-2.077	-3.566	3	76.896	0	1
F2173-1125	C18H14N4O5S	398.39	2-hydroxy-5-[(E)-2-{4-[(pyridin-2-yl)sulfamoyl]phenyl}diazen-1-yl]benzoic acid	1	8.25	20.914	14.074	2.456	-4.564	-2.559	-3.89	2	59.994	0	1
F2619-0022	C22H18N4O4S2	466.53	3-benzenesulfonamido-N-(6-acetamido-1,3-benzothiazol-2-yl)benzamide	3	11	27.972	20.002	2.441	-5.946	-1.954	-2.855	3	80.176	0	1
F5084-0852	C22H18N4O3S3	482.6	2-(2-{[2-(4-methoxyphenyl)-2-oxoethyl]sulfanyl}-1,3-thiazol-4-yl)-N-[4-(pyridin-3-yl)-1,3-thiazol-2-yl]acetamide	1	9.75	21.239	13.048	3.325	-3.462	-0.364	-1.499	3	100	0	1

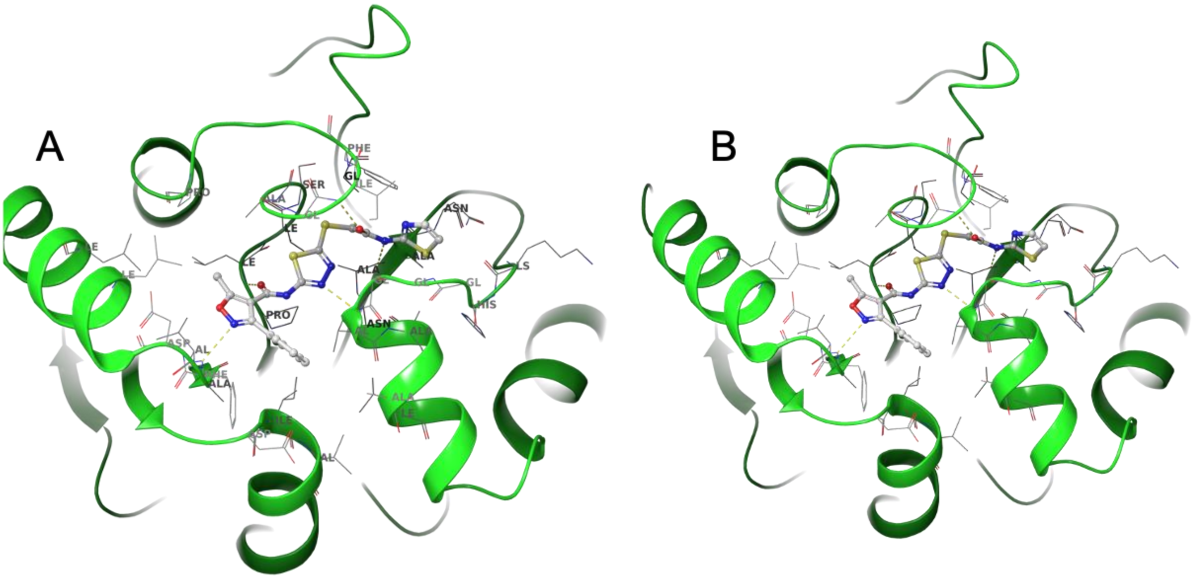

Docked Complex of Compound ID: F1877-0839

Figure S6. Docked complex of Compound ID: F1877-0839 WT Spike: ACE2 Binding (A) Labelled with Interacting residues (B) without interacting residues.

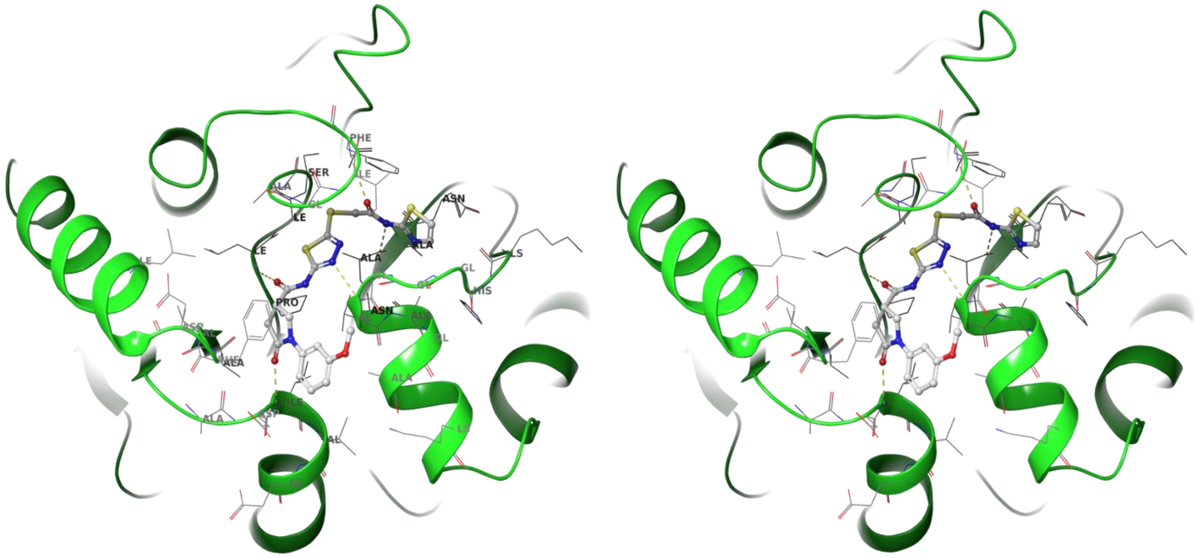

Docked Complex of Compound ID: F0470-0003

Figure S7. Docked complex of Compound ID: F0470-0003 WT Spike: ACE2 Binding (A) Labelled with Interacting residues (B) without interacting residues.

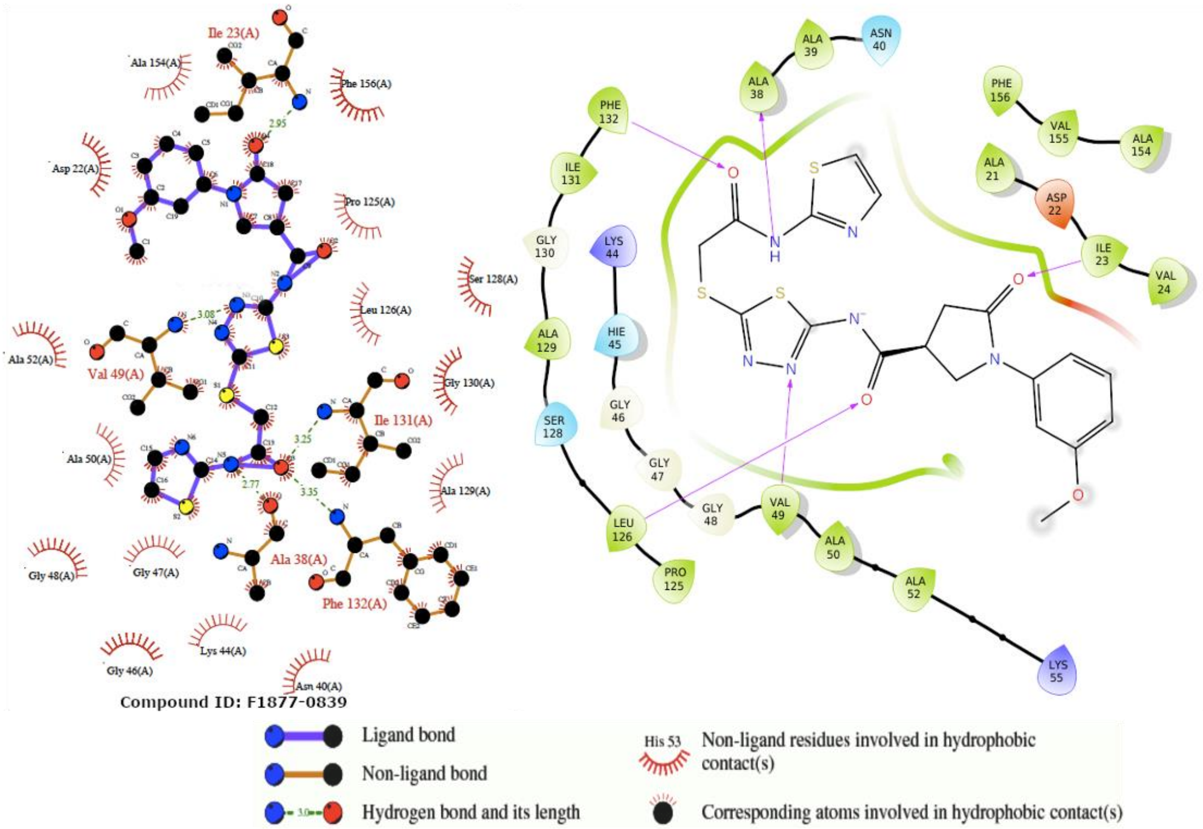

Figure S8. (Left): Ligplot Interactions of docked Complex of Compound ID: F1877-0839. (Right): schematic view of detailed ligand atom interactions with the protein residue.

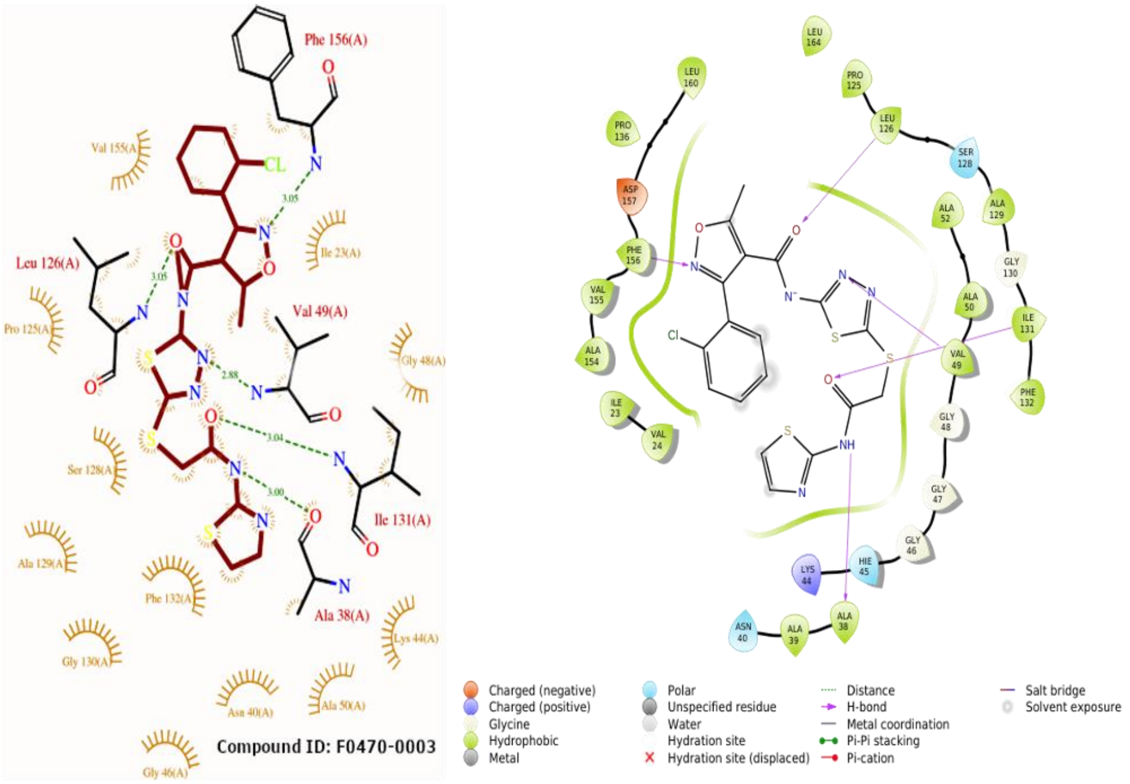

Figure S9. (Left): Ligplot Interactions of docked Complex of Compound ID: F0470-0003. (Right): schematic view of detailed ligand atom interactions with the protein residue.

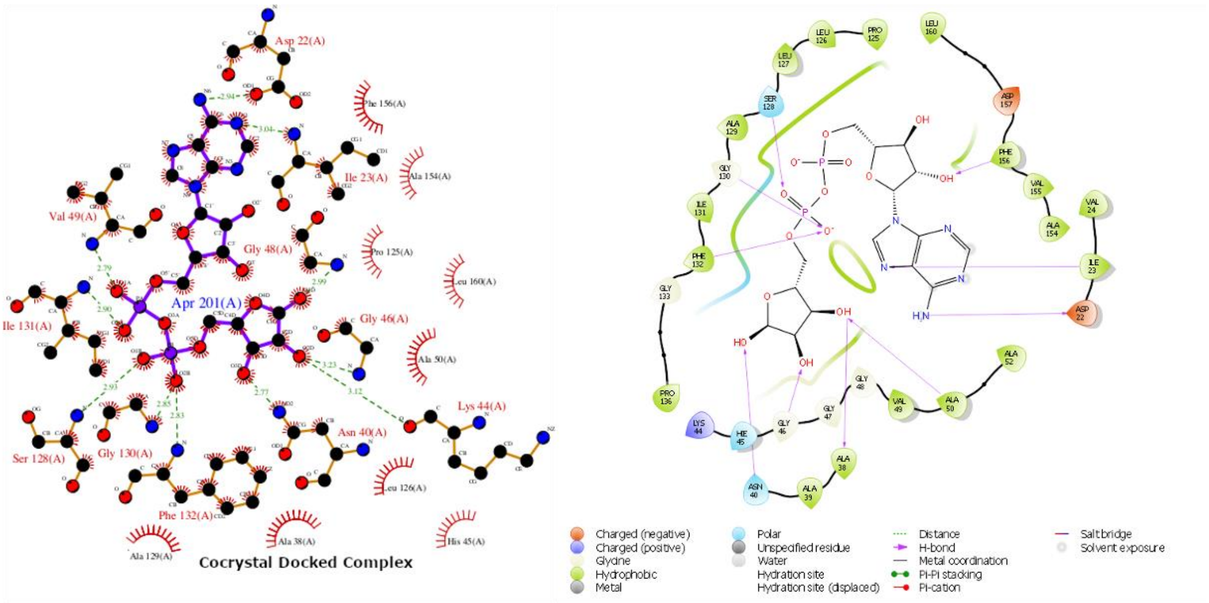

Figure S10. (Left): Ligplot Interactions of cocrystal Docked Complex. (Right): schematic view of detailed ligand atom interactions with the protein residue.
